# microRNAs and Their Targets in Apple (*Malus domestica* cv. “Fuji”) Involved in Response to Infection of Pathogen *Valsa mali*

**DOI:** 10.3389/fpls.2017.02081

**Published:** 2017-12-06

**Authors:** Hao Feng, Ming Xu, Xiang Zheng, Tongyi Zhu, Xiaoning Gao, Lili Huang

**Affiliations:** State Key Laboratory of Crop Stress Biology for Arid Areas and College of Plant Protection, Northwest A&F University, Yangling, China

**Keywords:** degradome sequencing, plant resistance, post-transcriptional regulation, small RNA, woody plant

## Abstract

miRNAs are important regulators involving in plant-pathogen interactions. However, their roles in apple tree response to Valsa canker pathogen (*Valsa mali, Vm*) infection were poorly understood. In this study, we constructed two miRNA libraries using the twig bark tissues of apple tree (*Malus domestica* Borkh. cv. “Fuji”) inoculated with *Vm* (IVm) and PDA medium (control, BMd). Among all detected miRNAs, 23 miRNAs were specifically isolated from BMd and 39 miRNAs were specifically isolated from IVm. Meanwhile, the expression of 294 miRNAs decreased; and another 172 miRNAs showed an increased expression trend in IVm compared with that in BMd. Furthermore, two degradome sequencing libraries were also constructed to identify the target genes of these miRNAs. In total, 353 differentially expressed miRNAs between IVm and BMd were detected to be able to target 1,077 unigenes with 2,251 cleavage sites. Based on GO and KEGG analysis, these genes were found to be mainly related to transcription regulation and signal transduction. In addition, we selected 17 miRNAs and 22 corresponding target genes to screen the expression profiles when apple twigs were infected by *Vm*. The expression trends of most miRNAs/target genes were consist with the results of deep sequencing. Many of them may involve in the apple twig-*Vm* interaction by inducing/reducing their expression. What's more, miRNAs and their target genes regulate the apple twig-*Vm* interaction by forming many complicated regulation networks rather than one to one model. It is worth that a conserved miRNAs mdm-miR482b, which was down regulated in IVm compared with BMd, has 14 potential target genes, most of which are disease resistance related genes. This indicates that mdm-miR482b may play important roles in apple twig response to *Vm*. More important, the feedback regulation of sRNA pathway in apple twig is also very complex, and play critical role in the interaction between apple twig and *Vm* based on the results of expression analysis. In all, the results will provide insights into the crucial functions of miRNAs in the woody plant, apple tree-*Vm* interaction.

## Introduction

As a class of conserved small RNAs in eukaryotes, microRNAs (miRNAs), with ~21–24 nucleotides (nt) in length, are generated from single-stranded RNA transcript with a self-complementary hairpin structure, and act as specific repressors of target gene expression through DNA methylation, histone modification, cleavage of the target transcript or inhibition of translation (Reinhart et al., 2002; Bartel, 2004; Ramachandran and Chen, 2009; Voinnet, 2009; Seo et al., 2013; Clancy et al., 2014). Since the first plant miRNA was discovered in 2002, miRNAs have been confirmed to act as powerful endogenous regulators in various biological processes, including developmental patterning, resistance to abiotic stresses and biotic stresses (Llave et al., 2002; Palatnik et al., 2003; Fujii et al., 2005; Navarro et al., 2006). Increasing evidences demonstrate that plant miRNAs are key players in plant-microbe interaction mechanisms (Jin, 2008; Padmanabhan et al., 2009; Weiberg et al., 2014; Thiebaut et al., 2015). However, most studies have focused on herbaceous plants, in particular, Arabidopsis, rice, wheat, etc. Regulation by sRNAs, particularly in response to pathogen infection, in economically important woody plants is poorly understood.

Apple (*Malus domestica* Borkh.) is one of the most widespread and economically important fruit trees worldwide. Specifically, in China, the apple planting area and production are nearly 2.26 million hectares and 40 million tons annually. The apple industry has been a pillar for rural economic development in northern China. However, the apple industry in China is suffering from huge economic losses due to the prevalence of various diseases. Among them, apple tree Valsa canker, caused by *Valsa mali* (*Vm*), is the major one (Wang et al., 2014). The pathogen causes decomposition of bark tissues of main trunk, lateral branch, and even leads to the death of twigs, branches, trunks, and eventually the entire tree. However, no immune or highly resistant variety to *Vm* has been reported. To date, the disease still cannot be effectively controlled, mainly because the information about the mechanism of apple tree in response to *Vm* was limited. Thus, accelerating studies on the mechanism of apple-*Vm* interaction will provide opportunities to increase the resistance to apple tree Valsa canker.

In the past few years, many studies about the pathogenic mechanism of *Vm* have been conducted at the genome level, transcriptome level, and functional genomics level (Ke et al., 2013; Hu et al., 2014; Yin et al., 2015). However, resistance mechanism of host in response to pathogen is not very clear. In 2016, Yin et al. (2016) reported that 2,713 apple genes were significantly up-regulated upon *Vm* infection, and they mainly focused on chitin, hormone and cell death biological processes. Because miRNAs are essential gene expression regulators, we hypothesize that they could be involved in the resistance response of apple tree to *Vm*. In fact, many miRNAs of apple have been isolated using both computational and high-throughput sequencing methods from different varieties and tissues, and these miRNAs have been confirmed to be related to tissue development (Gleave et al., 2008; Varkonyi-Gasic et al., 2010; Xia et al., 2012; Szczesniak and Makalowska, 2014; Qu et al., 2016; Xing et al., 2016). Moreover, many miRNAs were found to be involved in the biotic stress responses. In 2014, Yu et al. (2014) found that five apple miRNAs isolated from seedlings stems were responsive to apple ring rot infection. Ma et al. (2014) reported that the novel apple miRNA Md-miRLn11, isolated from a mixed sample of leaves, phloem, flower, and fruits, could affect the resistance to apple leaf spot disease by regulating a Md-NBS gene expression. In the next year, four apple miRNAs were isolated from the shoot tip tissues of four distinct rootstocks bench grafted with “Crimson Gala” scions, and presumed to be potentially important for resistance of apple trees to fire blight (Kaja et al., 2015). However, the specific miRNAs in the apple tree twig bark tissues, especially those involved in the interaction between the apple tree and *Vm*, are still not known.

In this study, high-throughput and degradome sequencing analyses were used to comprehensively identify the miRNAs and corresponding target genes from the apple twig bark tissues in response to *Vm*; these results will lay the foundation for exploration of the apple-*Vm* interaction mechanism at post-transcription level.

## Materials and methods

### Plant materials and samples collection

“Fuji” apple plants (*M. domestica* Borkh.) were grown in a greenhouse with 25°C and natural daylight conditions. The twigs were inoculated with *Vm* as described by Li et al. (2016). The samples of lesion borders were collected at 0, 12, and 48 h post-inoculation (hpi). Three individual biological repeats were prepared.

### RNA isolation, quantification, and qualification

According to the instruction of Trizol™ Reagent (Invitrogen, Carlsbad, CA), total RNA was extracted from each sample, and RNA purity was evaluated using NanoPhotometer® spectrophotometer (IMPLEN, CA, USA). RNA Nano 6000 Assay Kit and Agilent Bioanalyzer 2100 system (Agilent Technologies, CA, USA) were used to assess RNA concentration and integrity.

### Library construction and RNA deep sequencing

Three repeats of control samples and inoculation samples were mixed for small RNA library construction, respectively. Three micrograms of total RNA per sample was used as input material for the small RNA library. Small RNA library construction and RNA deep sequencing were proceeded following the detailed protocol provided by the genome sequencing company (Novogene, China).

### Bioinformatic analysis of sequence data

Raw data (raw reads) were first processed through custom Perl and Python scripts to obtain clean data. The clean data were mapped to the reference sequence in miRBase21.0 by Bowtie (Langmead et al., 2009), without mismatch to look for known miRNAs. Then, the other reads were integrated to predict novel miRNAs using the available software miREvo (Wen et al., 2010) and mirdeep2 (Friedlander et al., 2011). The miRNA counts as well as base bias were identified using custom scripts. The miFam.dat (http://www.mirbase.org/ftp.shtml) was used to look for families of known miRNAs. The novel miRNA precursor was submitted to Rfam (http://rfam.sanger.ac.uk/search/) to look for Rfam families.

### Venn diagrams of known miRNAs and novel miRNAs

Normalization formula (Normalized expression = Mapped read count/Total reads^*^1,000,000) was used to estimated miRNA expression levels (Zhou et al., 2010). DEGseq (2010) R package was used to analyze the differential expression of two samples with the criterion of *Q* < 0.01 and |log2(foldchange)| > 1 (Storey, 2003).

### Construction of degradome libraries

Target genes of candidate miRNAs were verified by degradome sequencing using total RNA same to the RNA used for small RNA sequencing library construction following the published parallel analysis of RNA Ends (PARE) protocol (German et al., 2009). The data analysis was processed following the procedure instructions (LC Sciences, Hangzhou, China).

### Target gene prediction and annotation for known and novel miRNAs

The psRobot_tarin psRobot was performed to predict target genes of miRNA (Wu et al., 2012). To further explore the detailed molecular mechanism of miRNAs in apple twig response to *Vm*, the target transcripts of differentially expressed miRNAs were analyzed by Gene Ontology Functional Annotation Suite (GO) and the Kyoto Encyclopedia of Genes and Genomes (KEGG). Subsequently, Revigo tool (http://revigo.irb.hr) was implemented for enrichment analysis of the target genes. The KOBAS software was used to test the statistical enrichment of the target gene candidates in the KEGG pathways (Mao et al., 2005).

### Expression analysis of miRNAs and their corresponding target genes

The expression of miRNAs in the apple twig bark tissues inoculated with *Vm* was determined using stem-loop RT-PCR technology (Feng et al., 2012). The expression analysis of the corresponding target genes was conducted as described by Feng et al. (2012). The apple translation elongation factor 1 alpha-subunit gene (EF-1a, Gene ID/Accession no. DQ341381) was selected to normalize the expression data of each miRNA and target gene. The relative expression of each miRNA and target gene was analyzed using the software of HemI (Heatmap Illustrator, version 1.0; Deng et al., 2014). All primers used in this work were listed in Table S12.

## Results

### Overview of the sRNA sequencing results

**Figure 1:**
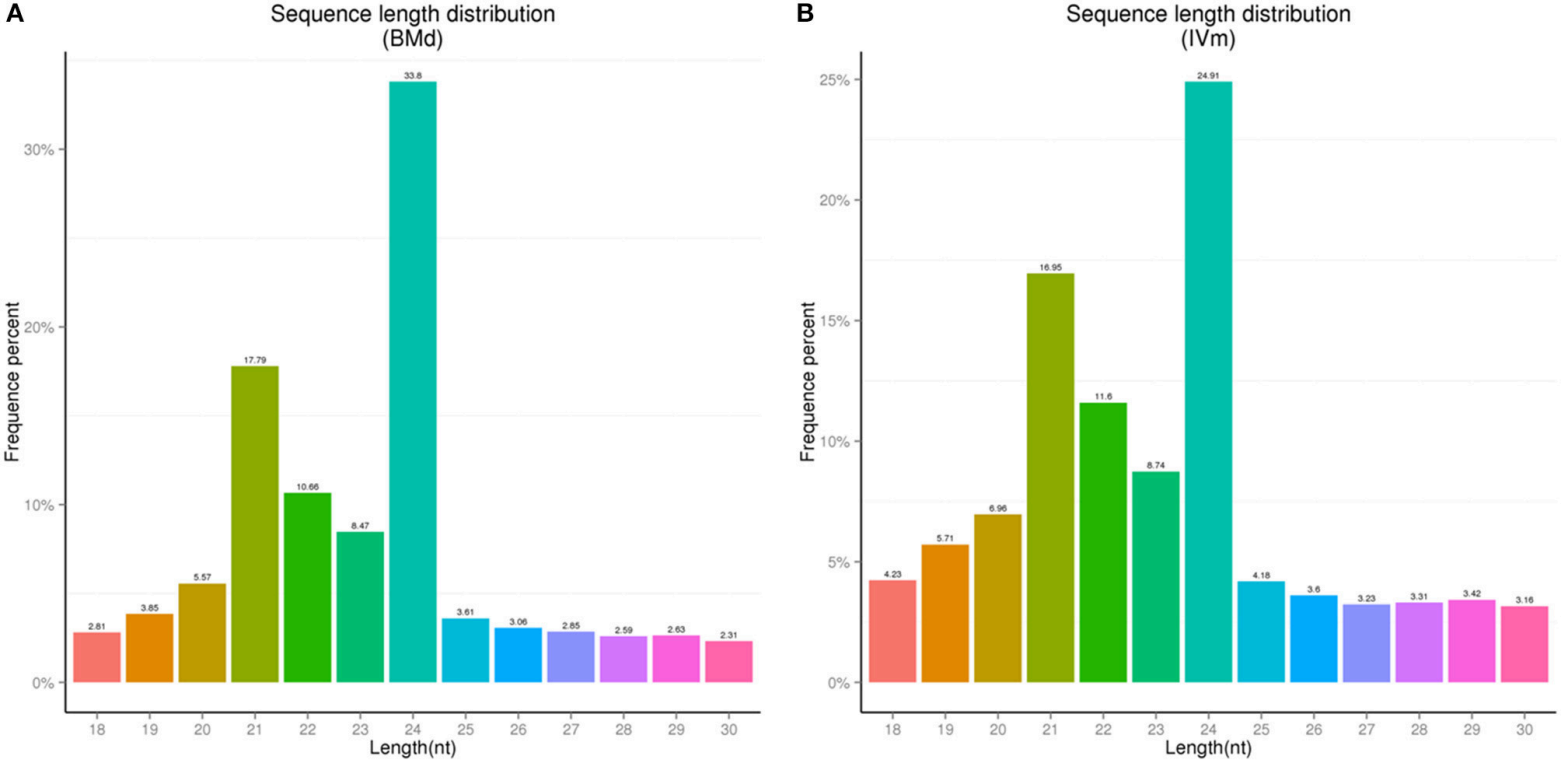
Length distribution of unique sequences in the two libraries from the apple twig bark. **(A)** BMd, the unique sequences were isolated from the apple twig bark as the mock control. **(B)** IVm, the unique sequences were isolated from the apple twig bark challenged with *V. mali* as the treatment sample.

Two sRNA libraries of apple twig (BMd and IVm) were constructed using Illumina high-throughput sequencing technology. In BMd, 13,065,359 raw reads (including Junk reads, Rfam, Repeats, valid reads, etc.) were generated, and 5,232,937 unique reads were isolated. Meanwhile, in IVm, 7,753,801 unique reads were isolated from 27,234,866 raw reads (Table S1). The proportion of different sRNAs for Rfam in BMd and IVm are shown in Figure S1. When the low-quality and junk sequences were removed, the left 3,491,357 valid reads in BMd and 4,949,663 valid reads in IVm were further analyzed, respectively. Among all sRNAs in both two libraries, 24 nt-length sRNAs were the most abundant, followed by 21, 22, and 23 nt-length. Despite this, an obvious difference existed between mock and treatment sample. For instance, the proportion of 24 nt-length sRNAs in BMd is 33.8%, whereas 24.9% in IVm. This difference was also found in other sRNAs with different length (Figure 1).

### Identification of miRNAs in apple twig bark

To explore all miRNAs in the bark of apple twig, we first compared all the valid reads in both BMd and IVm with known plant miRNAs in the miRBase to identify the conserved miRNA homologs. In total, 187 miRNAs of 46 families, generated from 138 miRNA precursors, were identified by mapping to the known miRNAs of apple. Meanwhile, a total of 37 miRNAs from 32 families, which could be mapped to miRNAs/pre-miRNAs of the selected species and the extended genome sequences from the mapped genome loci could form hairpin structures, were also preliminarily determined as candidate apple miRNAs. Moreover, we found 153 distinct reads could be mapped to the miRNAs/pre-miRNAs of the selected species in miRbase and the corresponding genome, but the extended sequences at the genome loci could not form hairpins, and another 51 reads could also be mapped to miRNAs/pre-miRNAs of the selected species in the miRbase, but the reads could not be mapped to the corresponding genome. In addition, 948 candidate miRNAs from 944 precursors, which could not be mapped to the pre-miRNAs of the selected species in miRbase were further determined as novel candidate miRNAs in apple twig bark, because they could be mapped to the corresponding genome, and the extended genome sequences from the genome may form hairpins (Table 1). Thus, we isolated 187 known miRNAs and 1189 candidate novel miRNAs from the apple twig bark tissues (Table S2). Most known miRNAs were conserved compared with those in other plants (Table S3; Figure S2). Moreover, the first nucleotide of these candidate miRNAs generally tended to be uracil (U) (Figure S3). All the raw data and clean data of these two libraries have been submitted to the GEO repository of NCBI (GEO accession: GSE104752), with the sample number GSM2807179 for BMd and GSM2807180 for IVm.

**Table 1:** Reads mapped to the selected miRNAs/pre-miRNAs in miRbase.

**Groups**	**Total**		**BMd**		**IVm**	
	**Pre-miRNA**	**miRNA**	**Pre-miRNA**	**miRNA**	**Pre-miRNA**	**miRNA**
gp1a	132	177	131	164	127	166
gp1b	6	10	6	9	4	8
gp2a	32	37	31	35	32	35
gp2b	155	153	149	146	155	149
gp3	47	51	35	38	44	47
gp4	944	948	942	945	944	948

*gp1a, Reads map to specific miRNAs/pre-miRNAs in miRbase and the pre-miRNAs further map to the genome & EST. gp1b, Reads map to selected (except for specific) miRNAs/pre-miRNAs in miRbase and the pre-miRNAs further map to the genome and EST. gp2a, Reads map to selected miRNAs/pre-miRNAs in miRbase. The mapped pre-miRNAs do not map to the genome, but the reads (and of course the miRNAs of the pre-miRNAs) map to genome. The extended genome sequences from the genome loci may form hairpins. gp2b, Reads were mapped to miRNAs/pre-miRNAs of selected species in miRbase and the mapped pre-miRNAs were not further mapped to genome, but the reads (and of course the miRNAs of the pre-miRNAs) were mapped to genome. The extended genome sequences from the genome loci may not form hairpins. gp3, Reads map to selected miRNAs/pre-miRNAs in miRbase. The mapped pre-miRNAs do not map to the genome, and the reads do not map to the genome. gp4, Reads do not map to selected pre-miRNAs in miRbase. But the reads map to genome and the extended genome sequences from genome may form hairpins*.

### Bio-informatic analysis of differentially expressed miRNAs in apple twig response to *Vm*

**Figure 2:**
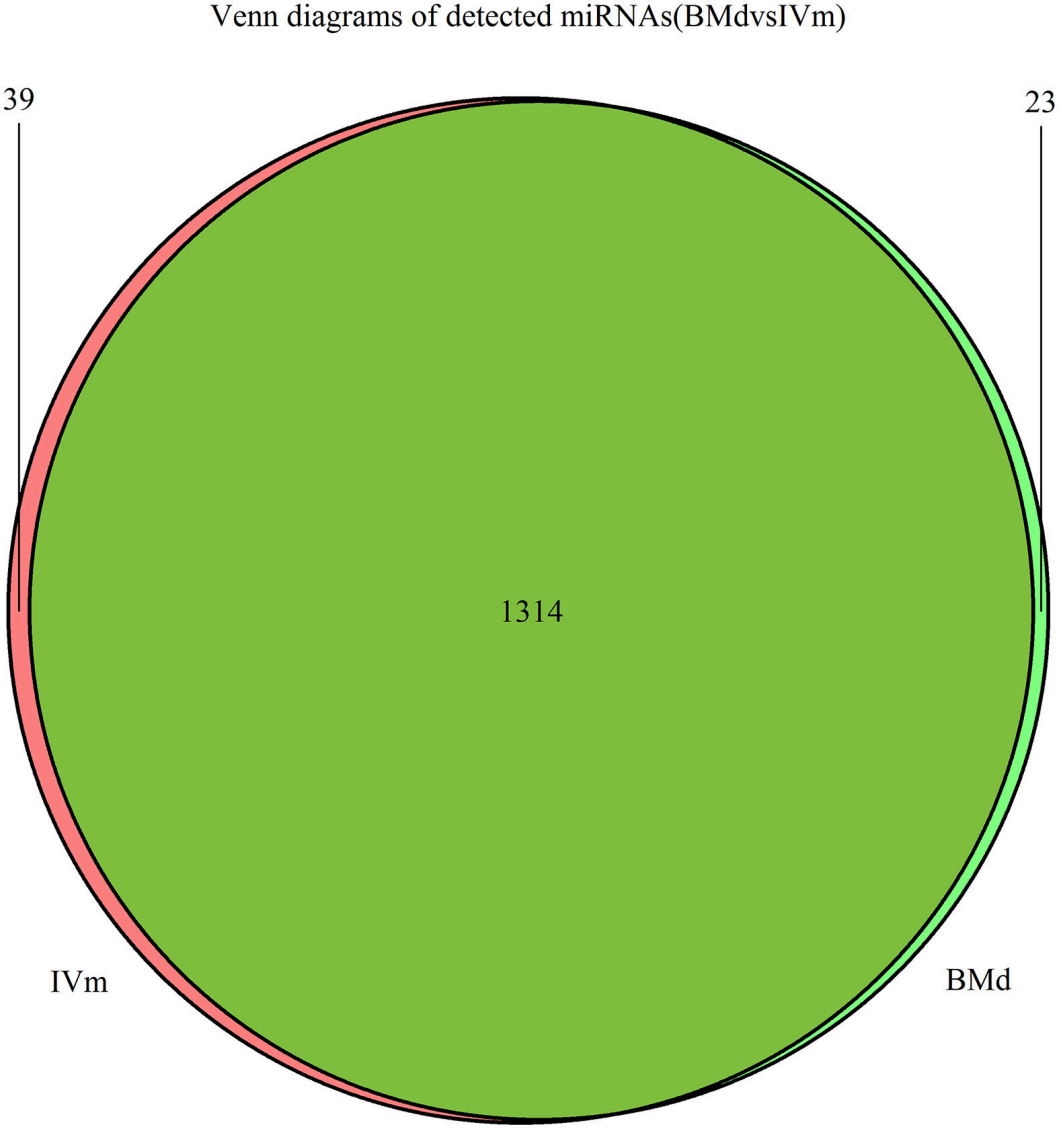
Venn diagram of the miRNAs in the apple twig bark in response to *V. mali* and the mock control. Venn diagram showing the miRNA candidates that are commonly expressed in the apple twig bark in response to *V. mali* (IVm) and the mock control (BMd) samples, as well as those expressed exclusively under one treatment but not the other.

**Figure 3:**
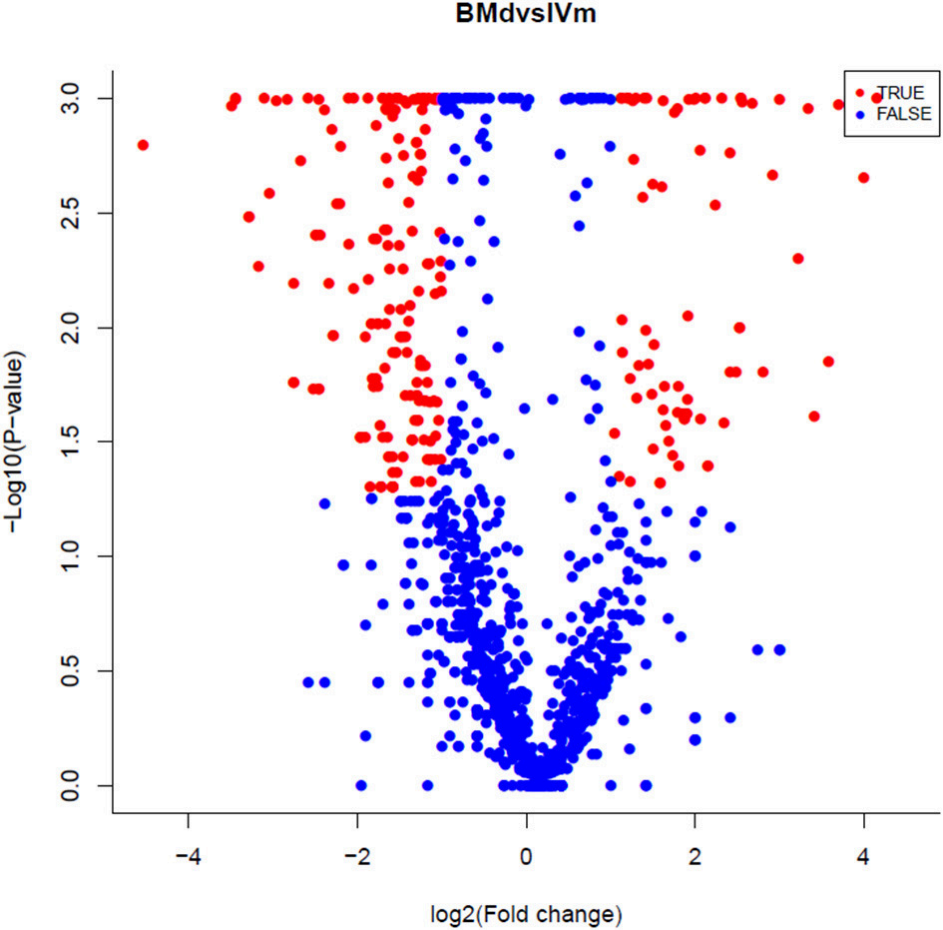
A volcano plot of the differentially expressed miRNAs in the apple twig bark challenged with *V. mali* compared with the mock control. For each miRNA, sequence reads were divided by the total sequence number, then multiplied by 1,000,000 (reads per million).

Specifically expressed miRNAs in apple twig inoculated with *Vm* were identified by comparing the miRNAs in IVm with those in BMd. Most miRNAs were co-expressed, with only 23 and 39 miRNAs expressed exclusively in BMd or IVm, respectively (Figure 2). The relative expression of miRNAs in *Vm*-infected apple twig compared with the mock-inoculated control was also analyzed with the reads' abundance. The samples showed various expression profiles when apple twig was challenged with *Vm* (Figure 3). Among them, 294 miRNAs were down-regulated when apple twigs were challenged with *Vm*, including 48 known apple miRNAs and 246 novel candidate miRNAs. Conversely, 172 miRNAs were up-regulated, including 36 known apple miRNAs and 136 novel candidate miRNAs (Table S4). In fact, only 113 and 40 candidate miRNAs showed down- and up-regulation in *Vm*-infected barks compared with the mock-inoculated control when the *p* ≤ 0.05 and |logfoldchange| ≥ 1 (Figure S4).

### Overview of the degradome sequencing results

To better understand the functions of miRNAs in apple twigs responding to *Vm* infection, the target genes of miRNAs in this study were determined using degradome sequencing technology. Two high-throughput degradome sequencing libraries (TBMd and TBVm) were constructed to determine the target genes of miRNA. A total of 2,251 cleavage sites from 1,077 unigenes were identified for 353 candidate miRNAs, including 158 known apple miRNAs. What's more, all of these genes have potential roles in pathogen responses (Table S5). For example, the target of a known apple miRNA, mdm-miR397a_L+1R+2_1ss22AG, was identified to be a nitrite reductase. Moreover, another important gene for disease resistance, Ca^2+^-transporting ATPase, was identified to be the target of another known apple miRNA, mdm-miR3627a_R-1. The target genes for some novel candidate miRNAs were also determined. The gene ppe-MIR172c-p5 was found to regulate the expression of monodehydroascorbate reductase, and PC-5p-154495_9 could affect the ABC transporters pathway (KEGG) by targeting ATP-binding cassette (Table 2). However, there were still large numbers of miRNAs whose targets remained undetected. All the results of these two libraries have also been submitted to the GEO repository of NCBI (GEO accession: GSE104752), with the sample number GSM2807181 for TBMd and GSM2807180 for TBVm.

**Table 2:** Typical target genes identified through degradome sequencing.

**SmallRNA**	**SmallRNA_seq**	**Transcript**	**Transcript Annotation**	**KEGG_pathways**	**Alignment Score**	**Alignment Range**	***P*-value**
mdm-miR403a	TTAGATTCACGCACAAACTCG	XM_008373619.1	Argonaute	–	1	3231–3251	0.00E+00
mdm-miR397a_L+1R+2_1ss22AG	ATTGAGTGCAGCGTTGATGAAGGT	XM_008365682.1	Nitrite reductase (NO-forming)	ko00910(Nitrogen metabolism)	2.5	738–760	0.00E+00
mdm-miR172a_R+1_1	AGAATCTTGATGATGCTGCAA	XM_008359912.1	AP2-like factor, euAP2 lineage	–	2	1715–1735	0.00E+00
mdm-miR396c_R-2	TTCCACAGCTTTCTTGAAC	XM_008372783.1	E3 ubiquitin-protein ligase	ko04120(Ubiquitin mediated proteolysis); ko04144(Endocytosis)	4	2420–2438	8.13E−42
mdm-miR3627a_R-1	TCGCAGGAGAGATGGCACT	XM_008393337.1	Ca^2+^-transporting ATPase	–	1.5	334–352	8.36E−76
mdm-miR162a	TCGATAAACCTCTGCATCCAG	XM_008360618.1	Endoribonuclease Dicer	–	2	3720–3741	2.00E−31
gma-MIR5368-p3_1ss3GA	CCAAGGGACAGTCTCAGGT	XM_008373238.1	Autophagy-related protein 7	ko04140(Regulation of autophagy)	3	946–964	3.22E−20
gma-MIR5368-p5_1ss2AT	GTGAGATACCACTCTGGA	XM_008360074.1	Cellulose synthase A	–	4	1825–1842	5.69E−09
ppe-MIR172c-p5	GCGGCATCATCAAGATTCAC	XM_008393540.1	Monodehydroascorbate reductase	ko00053(Ascorbate and aldarate metabolism)	4	518–536	1.92E−28
PC-3p-202837_7	AGGAATGTAAAATGAGATT	XM_008392258.1	Pyruvate dehydrogenase kinase	–	3.5	909–928	1.69E−19
PC-3p-370429_4	AGTGTAGAATGAGATTTTTTGAAG	XR_524420.1	Glutathione S-transferase	ko00980(Metabolism of xenobiotics by cytochrome P450);ko00982(Drug metabolism - cytochrome P450)	3	514–537	1.69E−08
PC-5p-154495_9	AGATGAATTTGAATTTTGAGATTT	XM_008355882.1	ATP-binding cassette	ko02010 (ABC transporters)	2.5	2608–2631	1.61E−29

### Target annotation of differentially expressed miRNAs in apple response to *Vm*

The GO annotation showed that these target genes could participate in various cellular processes, such as apoptosis, transcription regulation, auxin-mediated signaling pathway, ATP binding, DNA binding and protein binding activity (Table S6). To better explore the biological meaning, we further processed an enrichment analysis of target genes with *p* ≤ 0.01 using the Revigo tool. 106 genes were classified into three categories, and most them were linked into a complex net. Based on the results, the two most basic “Cellular Component” categories are “nucleus” and “SCF ubiquitin ligase complex” (Figure S5). In “Biological Process,” the top two are “auxin mediated signaling pathway” and “regulation of transcription, DNA-dependent” (Figure S6). And in “Molecular Function,” the two most categories are “protein dimerization activity” and “translation elongation factor activity” (Figure S7). Meanwhile, 124 KEGG pathways were classified, and the most common pathways were “Cysteine and methionine metabolism,” “Phenylpropanoid biosynthesis,” “Ribosome,” “Regulation of autophagy,” “basal transcription factors,” “Aminoacyl–tRNA biosynthesis,” “Nitrogen metabolism,” and “Glutathione metabolism” (Figure S8). We also found some pathways related particularly to disease response, such as plant-pathogen interaction, the MAPK signaling pathway, and ABC transporters (Table S7).

### *Vm* infection triggers the expression change of miRNAs and target genes

**Figure 4:**
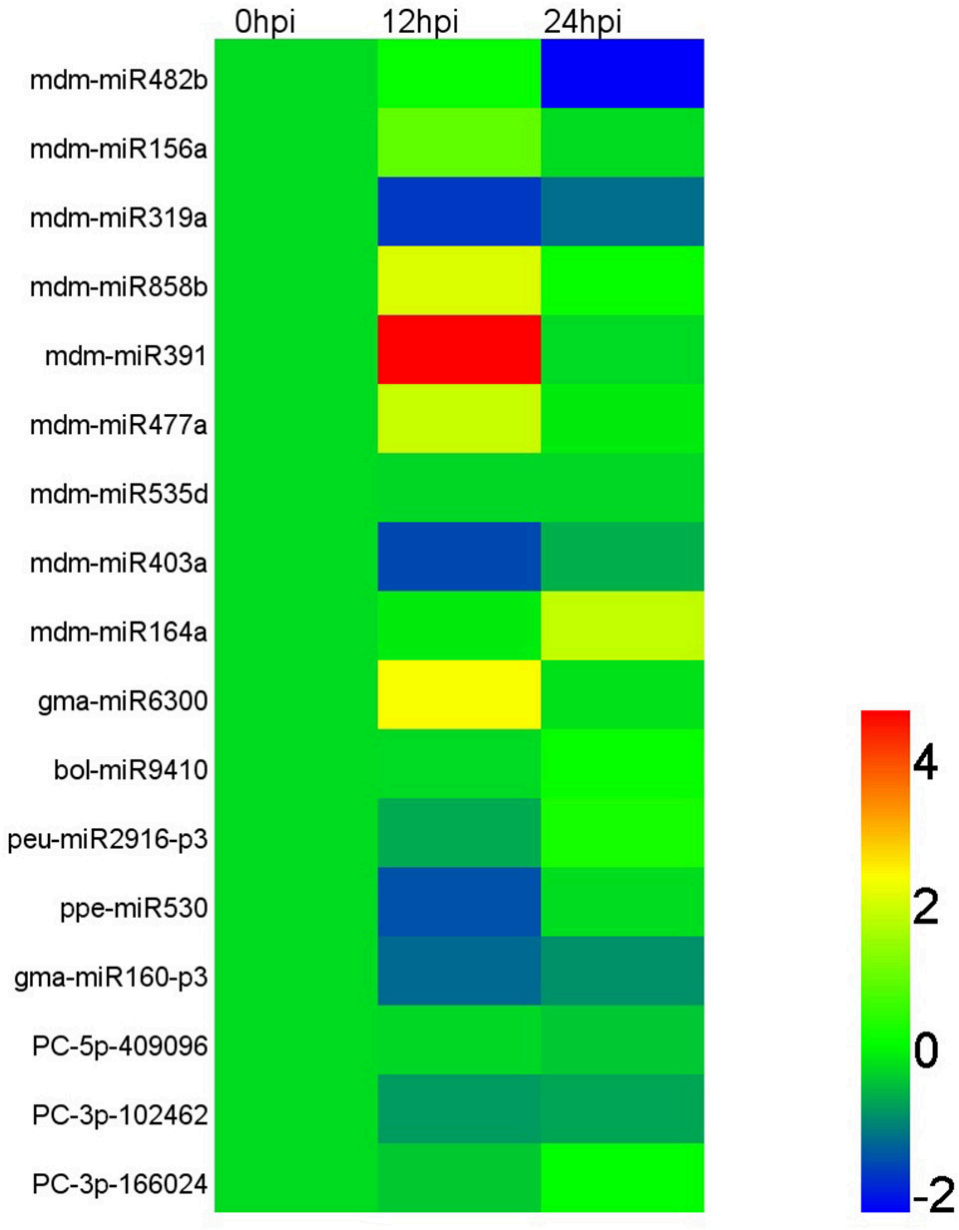
Relative expression of miRNAs in apple twig bark tissue—*V. mali* interaction. According to the sequencing read number, 17 miRNAs were selected for transcript accumulation analysis in apple twig bark tissue after challenge with *V. mali* (12 and 48 hpi). The relative expression level of the miRNAs in the *V. mali*-inoculated plants at each time point was calculated as the fold-change of the mock-inoculated plants at that time point using the comparative 2^−ΔΔ^CT method. The value of Log2 fold-change is used to construct the heat map. Different colors indicate the relative expression level of different miRNAs during the infection progress of *Vm* to apple twigs.

**Figure 5:**
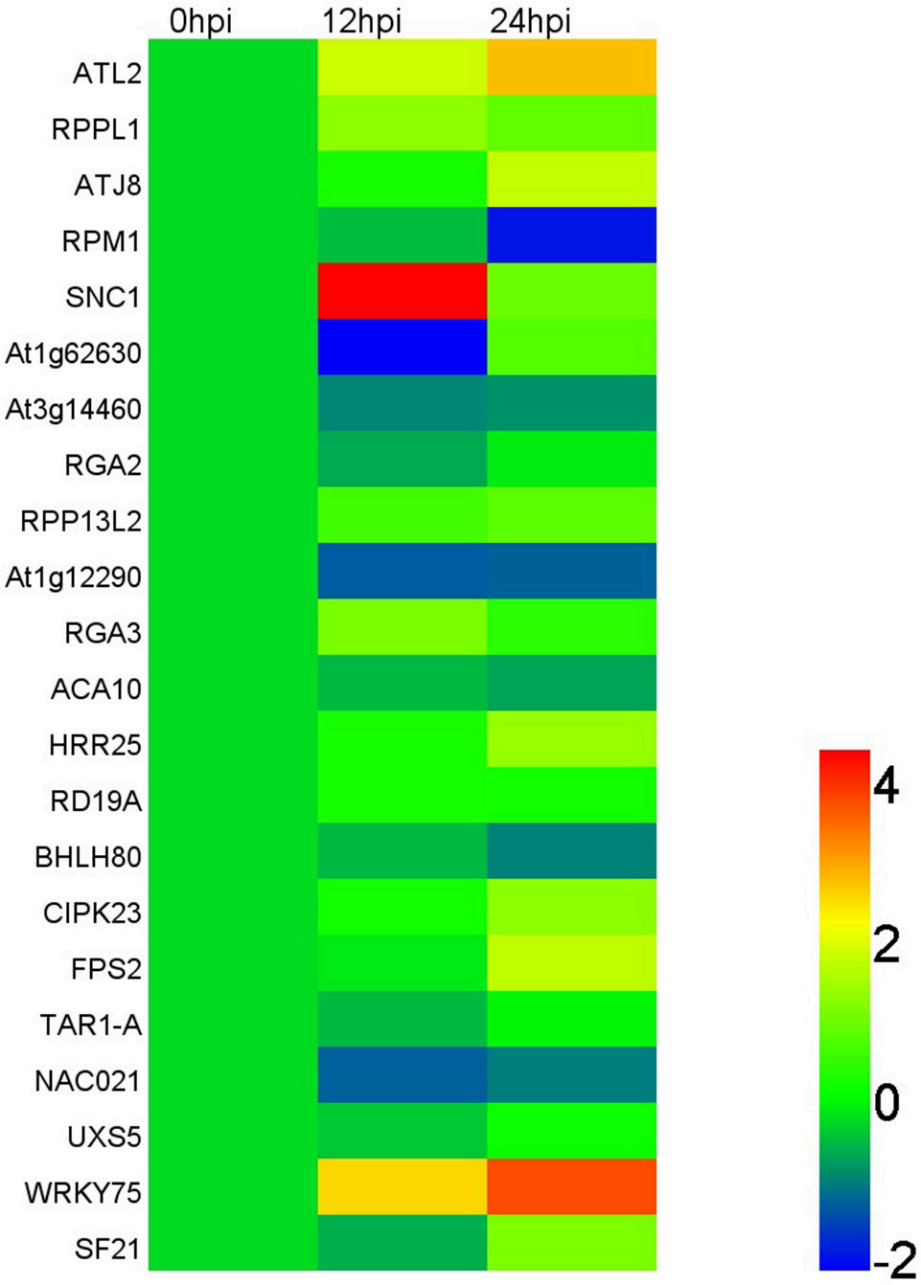
Relative expression of the target genes in apple twig bark tissue—*V. mali* interaction. Twenty-two identified targets of miRNAs were selected for transcript accumulation analysis in apple twig bark tissue after challenge with *V. mali* (12 and 48 hpi). The data were normalized to the expression level of apple translation elongation factor 1 alpha-subunit (EF). The relative expression level of the miRNAs in the *V. mali*-inoculated plants at each time point was calculated as the fold-change of the mock-inoculated plants at that time point using the comparative 2^−ΔΔ^CT method. The value of Log2 fold-change is used to construct the heat map. Different colors indicate the relative expression level of different target genes during the infection progress of *Vm* to apple twigs.

Based on the results of deep sequencing, lots of miRNAs were speculated to play roles in interaction between apple tree and *Vm*. And 17 miRNAs, which showed an obvious induced/reduced expression in IVm compared with BMd (*p* ≤ 0.05), together with more than 10 normalized reads number in IVm or BMd, were selected for qRT-PCR analysis to explore their expression in apple twig tissues response to *Vm* infection. Our results showed that mdm-miR156a, mdm-miR858b, mdm-miR391, mdm-miR477a, and gma-miR6300 were up-regulated during *Vm* infection process, and mdm-miR391 showed noticeable induction in the early stage of infection (hpi). Compared with the control, the expression levels of mdm-miR319a, mdm-miR403a, ppe-miR530, and gma-miR160-p3 were decreased. Moreover, mdm-miR482b, mdm-miR535d, bol-miR9410, peu-miR2916-p3, PC-5p-409096, PC-3p-102462, and PC-3p-166024 expressed stably during the entire infection progress of *Vm* (Figure 4; Table S8). Meanwhile, 22 identified targets, which were predicted to be important to stress response of plants and showed an obvious induced/reduced expression in TBVm compared with TBMd (*p* ≤ 0.05), were also selected for transcript analysis. Eleven genes were up-regulated when apple twigs were inoculated with *Vm*, and most of them were related to plant disease resistance, such as the homologs of ATL2, SNC1, WRKY75, RPPL1, and RGA3. Compared to the control, the expression of the homologs of RPM1, At1g62630, At1g12290, and NAC021 decreased, and the other seven genes showed stable expression during the entire infection progress of *Vm* (Figure 5; Table S9).

### miRNAs and corresponding target genes function by forming complex networks

**Figure 6:**
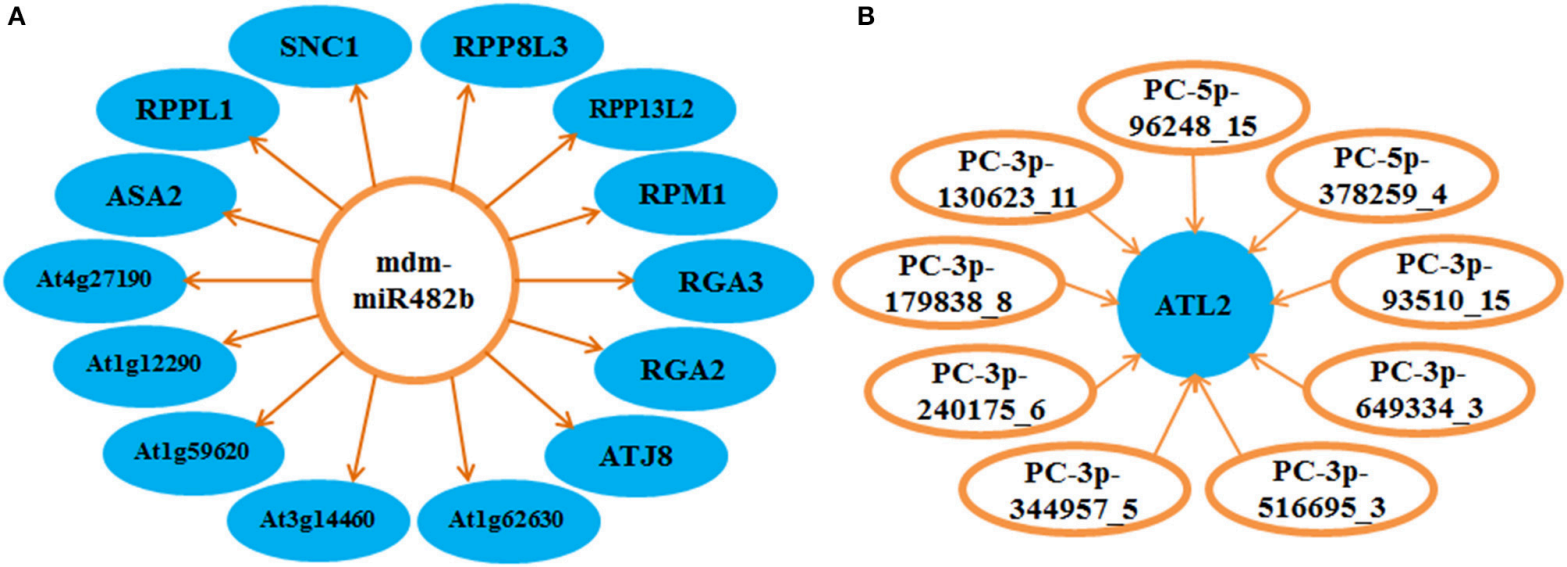
Disease resistance-related regulation network of miRNAs and corresponding targets in the interaction of apple twig bark and *V. mali*. Several miRNAs and their corresponding targets were selected to show the complex regulation net, and most of them were associated with plant disease resistance. **(A)** One miRNA was found to be able to regulate several targets. **(B)** One target could also be regulated by various miRNAs. ATL2, atlastin GTPase 2; ASA2, anthranilate synthase; At1g12290 and At1g59620, probable disease resistance protein; At3g14460 and RPM1, putative disease resistance protein; At1g62630 and At4g27190, disease resistance protein; ATJ8, J proteins; RGA2 and RGA3, Rho-type GTPase-activating protein; RPP13L2, putative disease resistance RPP13-like protein 2; RPP8L3, disease resistance RPP8-like protein 3; RPPL1, putative disease resistance RPP13-like protein 1; SNC1, TIR-NBS-LRR class disease resistance protein.

The regulatory mechanisms of miRNAs in the apple twig bark response to *Vm* were very complex. Based on the detected targets, we found that the one-to-one regulation model was not prevalent. Majority of miRNAs could target more than one transcript, and most miRNAs and their targets constructed a complex regulatory network. For example, ppe-MIR169i-p5_2ss1CT19GT was found to cleave 80 target transcripts, and the transcript XM_008348120.1 could be regulated by 29 candidate miRNAs (Tables S10, S11). Notably, the expression of atlastin GTPase 2 (ATL2) could be regulated by nine novel candidate miRNAs, and the conserved miRNA mdm-miR482b in apple could target 14 genes; most target genes were disease resistance related genes, such as the Rho-type GTPase-activating protein and the disease resistance protein family members (Figure 6). Moreover, these miRNAs/targets on the cross point may be essential in the disease response signal network.

### Feedback regulation of the sRNA pathway in apple-*Vm* interaction

**Figure 7:**
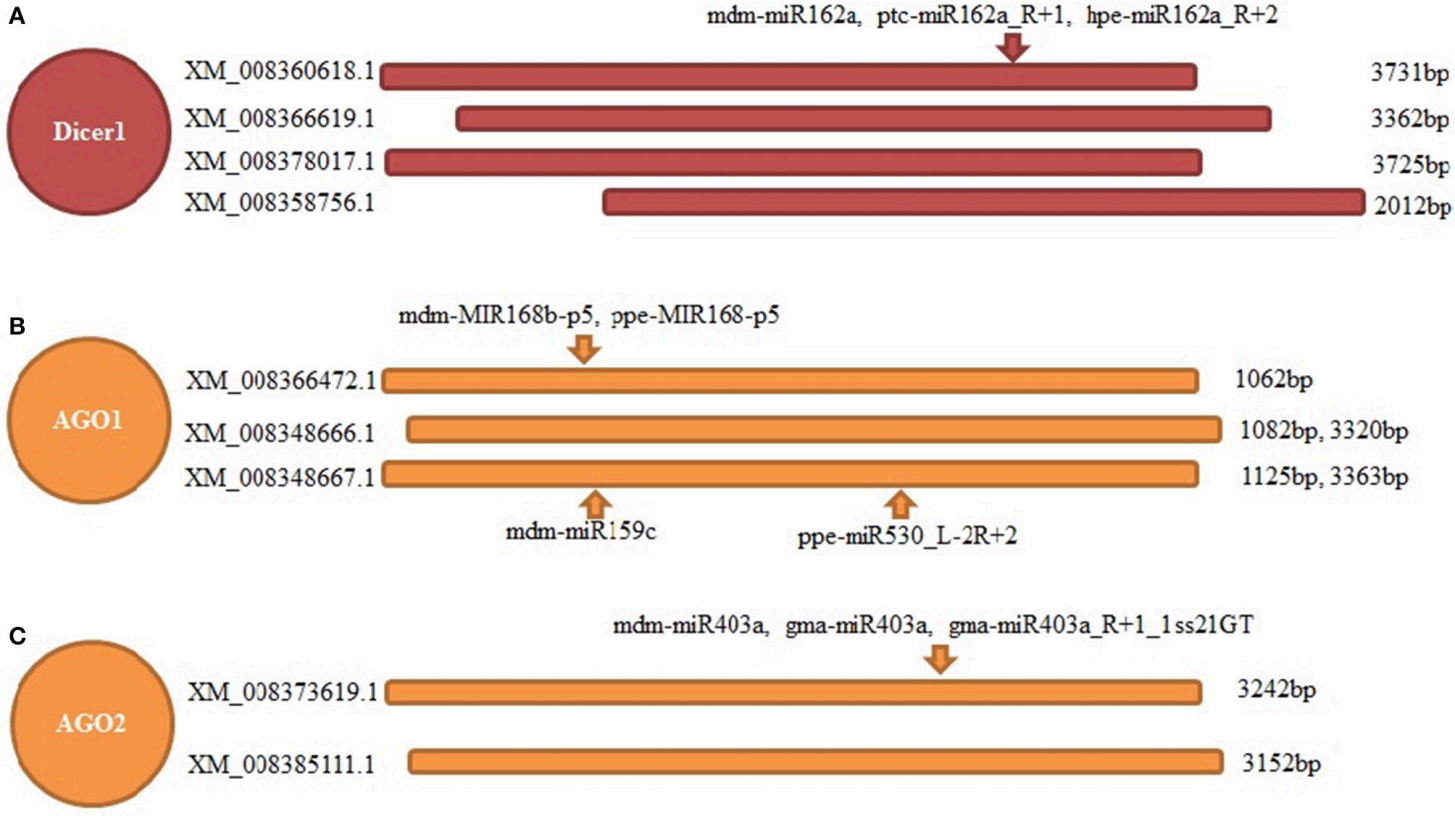
Feedback regulation of main components of RNAi pathway in apple twig bark—*V. mali* interaction. Three main components of RNAi pathway were found to be feedback regulated by several miRNAs. **(A)** Four transcripts of Dicer1 were detected to be cleaved at one common site by three miRNAs from the same family. **(B)** Three transcripts of AGO1 were detected to be cleaved at distinct sites by four miRNAs from three families. **(C)** Two transcripts of AGO2 were detected to be cleaved at one common site by three miRNAs from same family.

Dicer and AGO genes are the core components of the sRNA pathway; however, the role of miRNAs in their regulation remains unknown. Based on the results of degradome sequencing, we found four transcripts of the “switch” gene controlling miRNA generation (Dicer1), which could be cleaved by three different but highly homologous miRNAs from the same family of miR162. Moreover, two transcripts coding AGO2 were identified to have a cleavage site of three similar miRNAs from the miR403 family. Moreover, the important component, AGO1, which mainly binds miRNAs to cleave the corresponding targets, could be regulated by four miRNAs. Among them, two are from the miR168 family, and another two are from miR159 and miR530 families (Figure 7).

**Figure 8:**
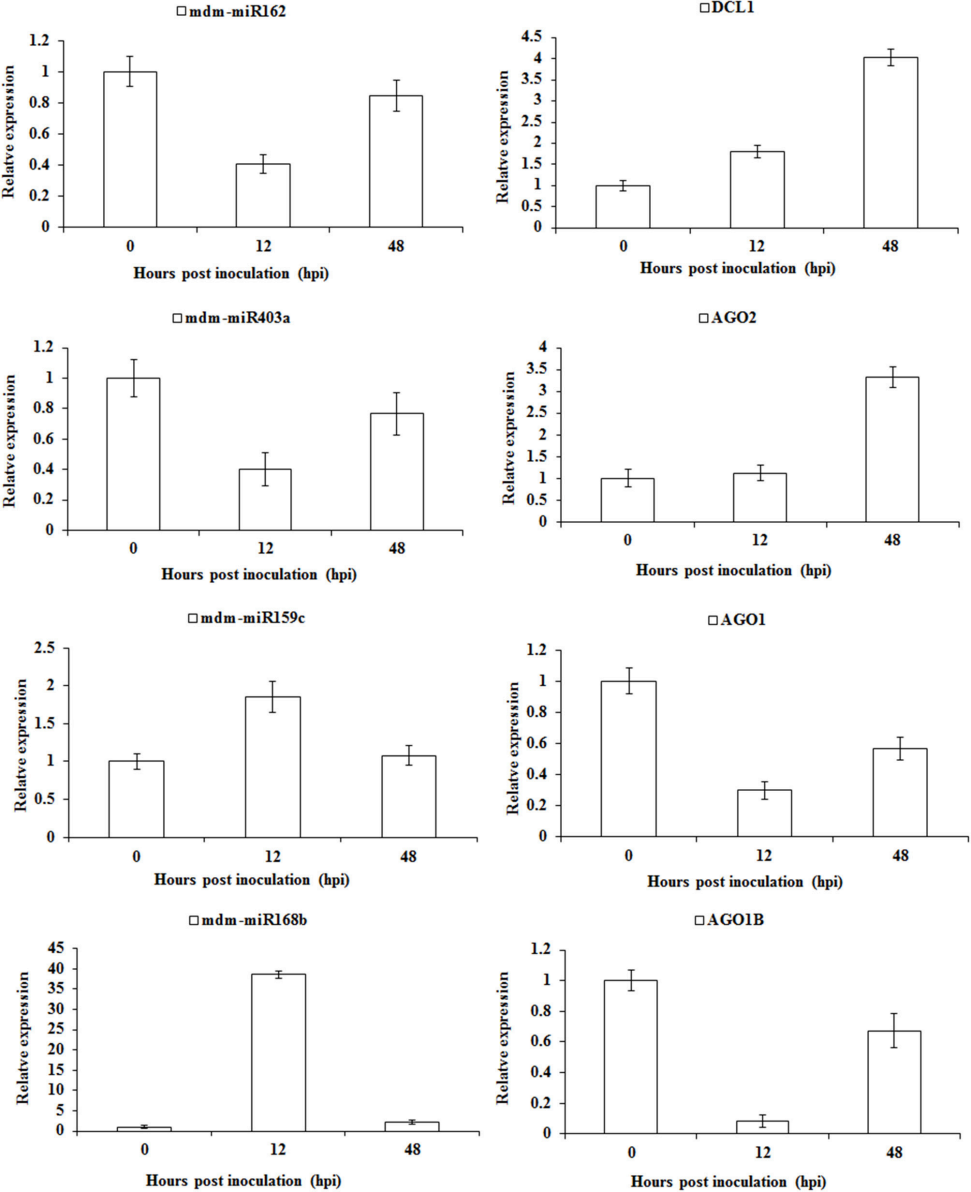
Relative transcription levels of miRNAs and corresponding target genes involved in the feedback regulation of sRNAs pathway in apple twig-*Vm* interaction. One DCL1, one AGO2, and two AGO1 transcripts were identified to be the target genes of mdm-miR162, mdm-miR403a, mdm-miR159c, and mdm-miR168b. Their transcript accumulation in apple twig bark tissues after challenge with *V. mali* was analyzed (12 and 48 hpi). The data were normalized to the inner reference gene apple translation elongation factor 1 alpha-subunit (EF-1a, Gene ID/Accession no. DQ341381). The mean value was calculated from three independent biological replicates. The relative expression level was analyzed as the fold-change of the mock-inoculated plants at that time point using the comparative 2^−ΔΔCT^ method.

To further verify the feedback regulation of the sRNA pathway in apple twig-*Vm* interaction, we detected the expression profiles for all these miRNAs and target genes (Figure 8). The gene mdm-miR162 was down-regulated at 12 hpi, while the expression of the corresponding target gene DCL1 began to increase and peaked at four-fold of the control. mdm-miR403a and AGO2 showed a similar expression trend during the *Vm* infection. However, mdm-miR159c and mdm-miR168b were increased, and the corresponding target gens, AGO1 and AGO1B, were down-regulated at 12 hpi, particularly the expression of mdm-miR168b and AGO1B, with ~40- and 10-fold changes compared with control, respectively. These results indicate that the feedback regulation of the sRNA pathway in apple twig was much more complex and critical. The detailed mechanism will be another interesting scientific question remaining to be explored.

## Discussion

As an abundant class of endogenous, non-coding small RNAs, miRNAs play important regulatory roles in organ development, phase change and defense responses, etc. (Bartel, 2004). Some apple miRNAs were identified using computational and/or sequencing approaches, and were found to be responsive to tissue development and biotic stresses response (Varkonyi-Gasic et al., 2010; Xia et al., 2012; Yu et al., 2014). However, there is no report of miRNAs involved in the disease response to *Vm*. Thus, the identification of apple miRNAs and the corresponding target genes in response to the *Vm* infection will help us to understand the regulatory mechanism of apple twig bark and *Vm* interaction.

In this study, we performed Illumina deep sequencing to construct two sRNAs libraries (BMd and IVm). The amount of 24 nt-length sRNAs in apple twig tissues was biggest in both BMd and IVm, which is consistent with the length distribution of sRNAs found in other apple tissues (Xia et al., 2012; Qu et al., 2016). Actually, 21 and 24 nt-length sRNAs were the main two sRNA classes among unique sequences based on previous reports on many other plants, such as wheat (Feng et al., 2017), rice (Morin et al., 2008), grapevine (Wang et al., 2011), tomato (Cao et al., 2014), and so on. According to the previous studies, 21 nt-length sRNAs are mainly related to mRNA cleavage (Hamilton and Baulcombe, 1999), while the 24 nt-length sRNAs are primarily involved in RNA-directed DNA methylation (Hamilton et al., 2002; Qi et al., 2006). For instance, it has been demonstrated that 24 nt-length sRNAs are associated with transcriptional gene silencing by targeting DNA methylation of three endogenous transposable elements in *Arabidopsis thaliana* (Lewsey et al., 2015). In wheat, 24 nt-length sRNAs were found to mainly match class I and class II transposable elements, which were also predicted to be more likely to be methylated (Cantu et al., 2010). But these results don't mean that 21 nt-length sRNAs were unrelated with DNA methylation. In subsequent studies, 21 nt-length sRNAs were confirmed to be involved in the establishment of DNA methylation, whereas the 21 nt-length species contributed to the amplification and maintenance of DNA methylation (Kim and Zilberman, 2014; Bond and Baulcombe, 2015). However, whether the roles of 24 nt-length miRNAs in plant-disease interactions are conducted through DNA methylation is still unknown. What's more, the proportion of same length sRNAs in BMd and IVm is also different in this study. In fungi and animals, there are more than two pathways to generate various sRNAs, with the main ways of Dicer-dependent and Dicer-independent (Aravin et al., 2006; Lee et al., 2010). In plant, there is no report to illustrate whether there is another way to generate sRNA like that. However, it has confirmed that many plant miRNAs expressed specially in different tissues or developmental stages, even response to the stresses response, thereby contributing to the specific target gene regulation (Inui et al., 2010). Actually, in our results, when the apple twig was challenged with *Vm*, the expression of key proteins in RNAi pathway changed a lot. Whether the difference of the proportion of same length sRNAs is associated with the expression change of these genes will still be explored in further study.

Plant miRNAs mainly regulate gene expression by cleaving mRNAs directly, and the cleavage normally occurs at the tenth nucleotide of the complementary region (Rhoades et al., 2002; Varkonyi-Gasic et al., 2010). Thus, the determination of corresponding target genes of miRNAs in apple twig bark is critical for functional exploration of the miRNAs. Degradome sequencing, as a high-throughput technology for target detection, has been used in several studies in the past few years (Addo-Quaye et al., 2008; Li et al., 2010; Yang et al., 2014; Yao et al., 2015). In this study, the target genes were also detected using this technology. To get more accurate results, gene mapping and annotation were referred to the transcriptomic information of apple twig bark challenged with *Vm*. As reported, many plant miRNAs play roles by targeting transcription factors or other regulatory/stress-response proteins (Ehrenreich and Purugganan, 2008; Guo et al., 2008; Varkonyi-Gasic et al., 2010; Zhang et al., 2013; Niu et al., 2016). In this study, the target genes of 84.5% of known apple miRNAs were determined, and many of them were associated with disease responses, such as Ca^2+^-transporting ATPase, aarF domain-containing kinase, E3 ubiquitin-protein ligase, and the mlo protein. What's more, most miRNAs and the corresponding target genes function by forming a complex interaction network. That means one miRNA could target several genes in different families, and one target gene could also be regulated by distinct miRNAs (German et al., 2009; Li et al., 2010; Lin et al., 2010). For example, mdm-miR482b could target 14 genes, and most these genes are CC-NBS-LRR class disease resistance proteins (At1g12290, At1g59620, At3g14460, At1g62630, At4g27190 and RPM1, RPP13L2, RPP8L3, and RPPL1) and a TIR-NBS-LRR class disease resistance protein (SNC1) (Bakker et al., 2006; Xu et al., 2014). In addition, mdm-miR482b could also regulate the expression of two Rho-type GTPase-activating proteins, which were critical regulator of calcium-dependent signal transduction, H_2_O_2_-mediated cell death, and so on (Wu et al., 2000). On the other hand, atlastin GTPase 2 (ATL2) was found to could be cleaved by nine novel candidate miRNAs. ATL2 was rapidly activated after incubation with elicitors of the pathogen response and was associated with the plant defense signaling pathways by ubiquitin ligases (Serrano and Guzmán, 2004). In 2016, miR863-3p was found to silence two negative regulators (*ARLPK1* and *ARLPK2*) of *Arabidopsis* defense at the early stage of infection of *Pseudomonas syringae* through mRNA degradation to promote immunity, and it could also silence SERRATE to positively regulate defense through translational inhibition at late infection stage (Niu et al., 2016). This result indicated that apple miRNAs might also play distinct roles by regulating the expression of the various target genes, possibly during the different developmental stages or the different stages of biological processes, such as the disease response. The expression of some selected miRNAs and target genes in apple twig bark in response to *Vm* were detected, and most were consistent with the results of sequencing-based estimations with up-/down-regulated expression trends. Combining the expression and the related reports, we speculated these regulated miRNAs/genes might contribute positively or negatively to the host-pathogen interaction.

Although many disease resistance-related miRNAs/genes were predicted and detected in this study, the host was still susceptible to *Vm* in reality. This indicated *Vm* might have evolved more “weapons” for attacking the host. Except for effectors, toxins, pectinases, and sRNAs from the pathogen could also enter the host cells to attack the host immunity by inhibiting the expression of critical genes in the disease response pathway, especially the sRNA pathway (Weiberg et al., 2013; Weiberg and Jin, 2015). For the sRNA pathway, short RNA duplexes are cleaved from long double-stranded RNAs by Dicer protein, and these sRNAs can guide gene silencing only when they are bound to the Argonaute proteins (AGOs) through a similar base-pairing mechanism with their target RNA transcripts (Zamore et al., 2000; Bernstein et al., 2001; Chapman and Carrington, 2007; Kim et al., 2010). In this study, we found that the feedback regulation of the sRNA pathway was more complex than that in the other plants. The induced expression of Dicer genes may result in a change of the quantity of different kind sRNAs. The results of differences in the sRNAs classes observed in mock and inoculated samples offered a good evidence to support it. Meanwhile, AGO1 seem to play critical role in this interaction. It was found that mdm-MIR168b-p5, ppe-MIR168-p5, mdm-miR159c, and ppe-miR530_L-2R+2 could all target AGO1 in apple. However, AGO1 in Arabidopsis was regulated by miR168 (Vaucheret et al., 2004). Therefore, the role of apple miRNAs in the orderly regulation of the sRNA pathway and the entry of the *Vm* sRNAs into the apple twig cells to attack the host sRNA pathway (by hijacking Dicer or AGO from the host sRNAs) to disturb the normal order of regulation of disease resistance are extremely interesting subjects for research.

In conclusion, candidate miRNAs and corresponding target genes involved in the apple-*Vm* interaction were detected using Illumina and degradome sequencing technology. Many stress responses related miRNAs/genes were isolated and the expression of them were further detected, particularly the cross point in signal network of disease resistance. Moreover, the feedback regulation of the key component proteins in sRNA pathway regulated by miRNAs was also found to be associated with the disease response. The sRNA pathway may have a mysterious function in the apple twig-*Vm* interaction, which should be further explored.

## Author contributions

HF and LH: contributed to the design of the work; HF and TZ: analyzed the sequencing data; MX and XG: drafted the work or revised it critically for important intellectual content; XZ: detected the expression of miRNAs and the corresponding target genes; LH: finally approved the version to be published, and was accountable for all aspects of the work in ensuring that questions related to the accuracy or integrity of any part of the work are appropriately investigated and resolved.

### Conflict of interest statement

The authors declare that the research was conducted in the absence of any commercial or financial relationships that could be construed as a potential conflict of interest.

## Supplementary material

Figure S1 Rfam sequence category of BMd and IVm.

Figure S2 Conservation profile of the identified miRNAs.

Figure S3 Nucleotide bias in each position of newly identified miRNAs.

Figure S4 Barplot of differentially expressed miRNAs between IVm and BMd.

Figure S5 Enrichment analysis of target genes in “Cellular Component” category.

Figure S6 Enrichment analysis of target genes in “Biological Process” category.

Figure S7 Enrichment analysis of target genes in “Molecular Function” category.

Figure S8 KEGG significant scatterplot between IVm and BMd.

Table S1 Overview of reads from raw data to cleaned sequences.

Table S2 All expressed miRNA in both IVm and BMd.

Table S3 Conservation of the identified miRNA with other species.

Table S4 Differentially expressed miRNAs between IVm and BMd.

Table S5 Degradome result in both IVm and BMd.

Table S6 GO plot statistics of target genes.

Table S7 KEGG pathway pics statistics in both IVm and BMd.

Table S8 Relative expression of miRNAs in apple twig bark tissue - *V. mali* interaction.

Table S9 Relative expression of target genes in apple twig bark tissue - *V. mali* interaction.

Table S10 Number statistics of target for each miRNA through predicting and degradome sequencing in both IVm and BMd.

Table S11 Number statistics of mRNA with miRNA targeting sites through predicting and degradome sequencing in both IVm and BMd.

Table S12 The primer sequences for quantitative real time PCR of candidate miRNAs and target genes.

## References

[B1] Addo-Quaye C. Eshoo T. W. Bartel D. P. Axtell M. J. (2008). Endogenous siRNA and miRNA targets identified by sequencing of the *Arabidopsis* degradome. Curr. Biol. 18, 758–762. 10.1016/j.cub.2008.04.042 18472421 PMC2583427

[B2] Aravin A. Gaidatzis D. Pfeffer S. Lagos-Quintana M. Landgraf P. Iovino N.. (2006). A novel class of small RNAs bind to MILI protein in mouse testes. Nature 442, 203–207. 10.1038/nature04916 16751777

[B3] Bakker E. G. Toomajian C. Kreitman M. Bergelson J. (2006). A genome-wide survey of R gene polymorphisms in *Arabidopsis*. Plant Cell 18, 1803–1818. 10.1105/tpc.106.042614 16798885 PMC1533970

[B4] Bartel D. P. (2004). MicroRNAs: genomics, biogenesis, mechanism, and function. Cell 116, 281–297. 10.1016/S0092-8674(04)00045-5 14744438

[B5] Bernstein E. Caudy A. A. Hammond S. M. Hannon G. J. (2001). Role for a bidentate ribonuclease in the initiation step of RNA interference. Nature 409, 363–366. 10.1038/35053110 11201747

[B6] Bond D. M. Baulcombe D. C. (2015). Epigenetic transitions leading to heritable, RNA-mediated de novo silencing in *Arabidopsis thaliana*. Proc. Natl. Acad. Sci. U.S.A. 112, 917–922. 10.1073/pnas.1413053112 25561534 PMC4311854

[B7] Cantu D. Vanzetti L. S. Sumner A. Dubcovsky M. Matvienko M. Distelfeld A.. (2010). Small RNAs, DNA methylation and transposable elements in wheat. BMC Genomics 11:408. 10.1186/1471-2164-11-408 20584339 PMC2996936

[B8] Cao X. Wu Z. Jiang F. Zhou R. Ynag Z. (2014). Identification of chilling stress-responsive tomato microRNAs and their target genes by high-throughput sequencing and degradome analysis. BMC Genomics 15:1130. 10.1186/1471-2164-15-1130 25519760 PMC4377850

[B9] Chapman E. J. Carrington J. C. (2007). Specialization and evolution of endogenous small RNA pathways. Nat. Rev. Genet. 8, 884–896. 10.1038/nrg2179 17943195

[B10] Clancy J. L. Patel H. R. Hussein S. M. I. Tonge P. D. Cloonan N. Corso A. J.. (2014). Small RNA changes en route to distinct cellular states of induced pluripotency. Nat. Commun. 5:5522. 10.1038/ncomms6522 25494340

[B11] Deng W. Wang Y. Liu Z. Cheng H. Xue Y. (2014). HemI: a toolkit for illustrating heatmaps. PLoS ONE 9:e111988. 10.1371/journal.pone.0111988 25372567 PMC4221433

[B12] Ehrenreich I. M. Purugganan M. D. (2008). Sequence variation of microRNAs and their binding sites in Arabidopsis. Plant Physiol. 146, 1974–1982. 10.1104/pp.108.116582 18305205 PMC2287364

[B13] Feng H. Huang X. L. Zhang Q. Wei G. R. Wang X. J. Kang Z. S. (2012). Selection of suitable inner reference genes for relative quantification expression of microRNA in wheat. Plant Physiol. Biochem. 51, 116–122. 10.1016/j.plaphy.2011.10.010 22153247

[B14] Feng H. Wang T. Feng C. Zhang Q. Zhang X. Huang L.. (2017). Identification of microRNAs and their corresponding targets involved in the susceptibility interaction of wheat response to *Puccinia striiformis* f. sp. tritici. Physiol. Plant. 157, 95–107. 10.1111/ppl.12407 26563616

[B15] Friedlander M. R. Mackowiak S. D. Li N. Chen W. Rajewsky N. (2011). miRDeep2 accurately identifies known and hundreds of novel microRNA genes in seven animal clades. Nucleic Acids Res. 40, 37–52. 10.1093/nar/gkr688 21911355 PMC3245920

[B16] Fujii H. Chiou T. J. Lin S. I. Aung K. Zhu J. K. (2005). A miRNA involved in phosphate-starvation response in *Arabidopsis*. Curr. Biol. 15, 2038–2043. 10.1016/j.cub.2005.10.016 16303564

[B17] German M. A. Luo S. Schroth G. Meyers B. C. Green P. J. (2009). Construction of parallel analysis of RNA ends (PARE) libraries for the study of cleaved miRNA targets and the RNA degradome. Nat. Protoc. 4, 356–362. 10.1038/nprot.2009.8 19247285

[B18] Gleave A. P. Ampomah-Dwamena C. Berthold S. Dejnoprat S. Karunairetnam S. Nain B. (2008). Identification and characterisation of primary microRNAs from apple (*Malus domestica* cv. Royal Gala) expressed sequence tags. Tree Genet. Genome 4, 343–358. 10.1007/s11295-007-0113-1

[B19] Guo X. Gui Y. Wang Y. Zhu Q. Helliwell C. Fan L. (2008). Selection and mutation on microRNA target sequences during rice evolution. BMC Genomics 9:454. 10.1186/1471-2164-9-454 18831738 PMC2567346

[B20] Hamilton A. J. Baulcombe D. C. (1999). A species of small antisense RNA in posttranscriptional gene silencing in plants. Science 286, 950–952. 10542148 10.1126/science.286.5441.950

[B21] Hamilton A. Voinnet O. Chappell L. Baulcombe D. (2002). Two classes of short interfering RNA in RNA silencing. EMBO J. 21, 4671–4679. 10.1093/emboj/cdf464 12198169 PMC125409

[B22] Hu Y. Dai Q. Liu Y. Yang Z. Song N. Gao X.. (2014). Agrobacterium tumefaciens-mediated transformation of the causative agent of valsa canker of apple tree *Valsa mali* var. mali. Curr. Microbiol. 68, 769–776. 10.1007/s00284-014-0541-8 24554343

[B23] Inui M. Martello G. Piccolo S. (2010). MicroRNA control of signal transduction. Nat. Rev. Mol. Cell Biol. 11, 252–263. 10.1038/nrm2868 20216554

[B24] Jin H. (2008). Endogenous small RNAs and antibacterial immunity in plants. FEBS Lett. 582, 2679–2684. 10.1016/j.febslet.2008.06.053 18619960 PMC5912937

[B25] Kaja E. Szcześniak M. W. Jensen P. J. Axtell M. J. McNellis T. Makałowska I. (2015). Identification of apple miRNAs and their potential role in fire blight resistance. Tree Genet. Genome 11:812 10.1007/s11295-014-0812-3

[B26] Ke X. Huang L. Han Q. Gao X. Kang Z. (2013). Histological and cytological investigations of the infection and colonization of apple bark by *Valsa mali* var. mali. Australas. Plant Pathol. 42, 85–93. 10.1007/s13313-012-0158-y

[B27] Kim M. Y. Zilberman D. (2014). DNA methylation as a system of plant genomic immunity. Trends Plant Sci. 19, 320–326. 10.1016/j.tplants.2014.01.014 24618094

[B28] Kim Y. K. Heo I. Kim V. N. (2010). Modifications of small RNAs and their associated proteins. Cell 143, 703–709. 10.1016/j.cell.2010.11.018 21111232

[B29] Langmead B. Trapnell C. Pop M. Salzberg S. L. (2009). Ultrafast and memory-efficient alignment of short DNA sequences to the human genome. Genome Biol. 10:R25. 10.1186/gb-2009-10-3-r25 19261174 PMC2690996

[B30] Lee H. C. Li L. Gu W. Xue Z. Crosthwaite S. K. Pertsemlidis A.. (2010). Diverse pathways generate microRNAs-like RNAs and dicer-independent small interfering RNAs in fungi. Cell 38, 803–814. 10.1016/j.molcel.2010.04.005 20417140 PMC2902691

[B31] Lewsey M. G. Hardcastle T. J. Melnyk C. W. Molnar A. Valli A. Urich M. A.. (2015). Mobile small RNAs regulate genome-wide DNA methylation. Proc. Natl. Acad. Sci. U.S.A. 113, 801–810. 10.1073/pnas.1515072113 26787884 PMC4760824

[B32] Li Y. F. Zheng Y. Addo-Quaye C. Zhang L. Saini A. Jagadeeswaran G.. (2010). Transcriptome-wide identification of microRNA targets in rice. Plant J. 62, 742–759. 10.1111/j.1365-313X.2010.04187.x 20202174

[B33] Li Z. Gao X. Kang Z. Huang L. (2016). *Saccharothrix yanglingensis* strain Hhs.015 is a promising biocontrol agent on apple Valsa canker. Plant Dis. 100, 510–514. 10.1094/PDIS-02-15-0190-RE 30694140

[B34] Lin S. I. Santi C. Jobet E. Lacut E. El Kholti N. Karlowski W. M.. (2010). Complex regulation of two target genes encoding SPX-MFS proteins by rice miR827 in response to phosphate starvation. Plant Cell Physiol. 51, 2119–2131. 10.1093/pcp/pcq170 21062869

[B35] Llave C. Xie Z. Kasschuau K. D. Carrington J. C. (2002). Cleavage of scarecrow-like mRNA targets directed by a class of Arabidopsis miRNA. Science 297, 2053–2056. 10.1126/science.1076311 12242443

[B36] Ma C. Lu Y. Bai S. Zhang W. Duan X. Meng D.. (2014). Cloning and characterization of miRNAs and their targets, including a novel miRNA-targeted NBS-LRR protein class gene in apple (*Golden Delicious*). Mol. Plant 7, 218–230. 10.1093/mp/sst101 23880633

[B37] Mao X. Cai T. Olyarchuk J. G. Wei L. (2005). Automated genome annotation and pathway identification using the KEGG orthology (KO) as a controlled vocabulary. Bioinformatics 21, 3787–3793. 10.1093/bioinformatics/bti430 15817693

[B38] Morin R. D. Aksay G. Dolgosheina E. Ebhardt H. A. Magrini V. Mardis E. R.. (2008). Comparative analysis of the small RNA transcriptomes of *Pinus contorta* and *Oryza sativa*. Genome Res. 18, 571–584. 10.1101/gr.6897308 18323537 PMC2279245

[B39] Navarro L. Dunoyer P. Jay F. Arnold B. Dharmasiri N. Estelle M.. (2006). A plant miRNA contributes to antibacterial resistance by repressing auxin signaling. Science 312, 436–439. 10.1126/science.1126088 16627744

[B40] Niu D. Lii Y. E. Chellappan P. Lei L. Peralta K. Jiang C. (2016). miRNA863-3p sequentially targets negative immune regulator pseudokinases ARLPKs and positive regulator SERRATE upon bacterial infection. Nat. Commun. 7:11324 10.1038/ncomms11324 27108563 PMC4848489

[B41] Padmanabhan C. Zhang X. Jin H. (2009). Host small RNAs are big contributors to plant innate immunity. Curr. Opin. Plant Biol. 12, 465–472. 10.1016/j.pbi.2009.06.005 19608454

[B42] Palatnik J. F. Allen E. Wu X. Schommer C. Schwab R. Carrington J. C.. (2003). Control of leaf morphogenesis by microRNAs. Nature 425, 257–263. 10.1038/nature01958 12931144

[B43] Qi Y. He X. Wang X. J. Kohany O. Jurka J. Hannon G. J. (2006). Distinct catalytic and non-catalytic roles of ARGONAUTE4 in RNA-directed DNA methylation. Nature 443, 1008–12012. 10.1038/nature05198 16998468

[B44] Qu D. Yan F. Jiang X. Yang H. Gao Z. Dong Y. (2016). Identification of microRNAs and their targets associated with fruit-bagging and subsequent sunlight re-exposure in the “Grannu Smith” apple exocarp using high-throughput sequencing. Front. Plant Sci. 7:27 10.3389/fpls.2016.00027 26870053 PMC4734179

[B45] Ramachandran V. Chen X. (2009). Small RNA metabolism in *Arabidopsis*. Trends Plant Sci. 13, 368–374. 10.1016/j.tplants.2008.03.008 18501663 PMC2569976

[B46] Reinhart B. J. Weinstein E. G. Rhoades M. W. Bartel B. Bartel D. P. (2002). MicroRNAs in plants. Genes Dev. 16, 1616–1626. 10.1101/gad.1004402 12101121 PMC186362

[B47] Rhoades M. W. Reinhart B. J. Lim L. P. Burge C. B. Bartel B. Bartel D. P. (2002). Prediction of plant microRNA targets. Cell 110, 513–520. 10.1016/S0092-8674(02)00863-2 12202040

[B48] Seo G. J. Kincaid R. P. Phanaksri T. Burke J. M. Pare J. M. Cox J. E.. (2013). Reciprocal inhibition between intracellular antiviral signaling and the RNAi machinery in mammalian cells. Cell Host Microbe 14, 435–445. 10.1016/j.chom.2013.09.002 24075860 PMC3837626

[B49] Serrano M. Guzmán P. (2004). Isolation and gene expression analysis of *Arabidopsis thaliana* mutants with constitutive expression of ATL2, an early elicitor-response RING-H2 zinc-finger gene. Genetics 167, 919–929. 10.1534/genetics.104.028043 15238540 PMC1470891

[B50] Storey J. D. (2003). The positive false discovery rate: a Bayesian interpretation and the q-value. Ann. Stat. 31, 2013–2035. 10.1214/aos/1074290335

[B51] Szczesniak M. W. Makalowska I. (2014). miRNEST 2.0: a database of plant and animal microRNAs. Nucleic Acids Res. 42, 74–77. 10.1093/nar/gkt1156 24243848 PMC3965105

[B52] Thiebaut F. Grativol C. Hemerly A. S. Ferreira P. C. G. (2015). MicroRNA networks in plant-microorganism interactions. Trop. Plant Biol. 8, 40–50. 10.1007/s12042-015-9149-9

[B53] Varkonyi-Gasic E. Gould N. Sandanayaka M. Sutherland P. MacDiarmid R. M. (2010). Characterisation of microRNAs from apple (*Malus domestica* 'Royal Gala') vascular tissue and phloem sap. BMC Plant Biol. 10:159. 10.1186/1471-2229-10-159 20682080 PMC3095296

[B54] Vaucheret H. Vazquez F. Crété P. Bartel D. P. (2004). The action of ARGONAUTE1 in the miRNA pathway and its regulation by the miRNA pathway are crucial for plant development. Gene Dev. 18, 1187–1197. 10.1101/gad.1201404 15131082 PMC415643

[B55] Voinnet O. (2009). Origin, biogenesis, and activity of plant microRNAs. Cell 136, 669–687. 10.1016/j.cell.2009.01.046 19239888

[B56] Wang C. Wang X. C. Kibeta N. K. Song C. N. Zhang C. Q. Li X. Y.. (2011). Deep sequencing of grapevine flower and berry short RNA library for discovery of novel microRNAs and validation of precise sequences of grapevine microRNAs deposited in miRBase. Physiol. Plant. 143, 64–81. 10.1111/j.1399-3054.2011.01481.x 21496033

[B57] Wang X. L. Zang R. Yin Z. Kang Z. Huang L. (2014). Delimiting cryptic pathogen species causing apple Valsa canker with multilocus data. Ecol. Evol. 4, 1369–1380. 10.1002/ece3.1030 24834333 PMC4020696

[B58] Weiberg A. Jin H. (2015). Small RNAs-the secret agents in the plant–pathogen interactions. Curr. Opin. Plant Biol. 26, 87–94. 10.1016/j.pbi.2015.05.033 26123395 PMC4573252

[B59] Weiberg A. Wang M. Bellinger M. Jin H. (2014). Small RNAs: a new paradigm in plant-microbe interactions. Ann. Rev. Phytopathol. 52, 495–516. 10.1146/annurev-phyto-102313-045933 25090478

[B60] Weiberg A. Wang M. Lin F.-M. Zhao H. Zhang Z. Kaloshian I.. (2013). Fungal small RNAs suppress plant immunity by hijacking host RNA interference pathways. Science 342, 118–123. 10.1126/science 24092744 PMC4096153

[B61] Wen M. Shen Y. Shi S. Tang T. (2010). miREvo: an integrative microRNA evolutionary analysis platform for next-generation sequencing experiments. BMC Bioinformatics 13:140. 10.1186/1471-2105-13-140 22720726 PMC3410788

[B62] Wu G. Li H. Yang Z. (2000). Arabidopsis RopGAPs are a novel family of Rho GTPase-activating proteins that require the Cdc42/Rac-interactive binding motif for Rop-specific GTPase stimulation. Plant Physiol. 124, 1625–1636. 10.1104/pp.124.4.1625 11115880 PMC59861

[B63] Wu H. J. Ma Y. K. Chen T. Wang M. Wang X. J. (2012). PsRobot: a web-based plant small RNA meta-analysis toolbox. Nucleic Acids Res. 40, 22–28. 10.1093/nar/gks554 22693224 PMC3394341

[B64] Xia R. Zhu H. An Y.-Q. Beers E. P. Liu Z. (2012). Apple miRNAs and tasiRNAs with novel regulatory networks. Genome Biol. 13:R47. 10.1186/gb-2012-13-6-r47 22704043 PMC3446319

[B65] Xing L. Zhang D. Zhao C. Li Y. Ma J. An N.. (2016). Shoot bending promotes flower bud formation by miRNA-mediated regulation in apple (*Malus domestica* Borkh.). Plant Biotechnol. J. 14, 749–770. 10.1111/pbi.12425 26133232 PMC4755197

[B66] Xu F. Cheng Y. Kapos P. Huang Y. Li X. (2014). P-loop-dependent NLR SNC1 can oligomerize and activate immunity in the nucleus. Mol. Plant 7, 1801–1804. 10.1093/mp/ssu097 25237053

[B67] Yang K. Rong W. Qi L. Li J. Wei X. Zhang Z. (2014). Isolation and characterization of a novel wheat cysteine-rich receptor-like kinase gene induced by *Rhizoctonia cerealis*. Sci. Rep. 3:3021. 10.1038/srep03021 24149340 PMC3805973

[B68] Yao F. Zhu H. Yi C. Qu H. Jiang Y. (2015) MicroRNAs targets in senescent litchi fruit during ambient storage post-cold storage shelf life. BMC. Plant. Biol. 15:181. 10.1186/s12870-015-0509-2 26179282 PMC4504174

[B69] Yin Z. Liu H. Li Z. Ke X. Dou D. Gao X.. (2015) Genome sequence of *Valsa* canker pathogens uncovers a potential adaptation of colonization of woody bark. New Phytol. 208, 1202–1216. 10.1111/nph.13544 26137988

[B70] Yin Z. Ke X. Kang Z. Huang L. (2016). Apple resistance response against *Valsa mali* revealed by transcriptomics analyses. Physiol. Mol. Plant Pathol. 93, 85–92. 10.1016/j.pmpp.2016.01.004

[B71] Yu X. Y. Du B. B. Gao Z. H. Zhang S. J. Tu X. T. Chen X. Y.. (2014). Apple ring rot-responsive pupative microRNAs revealed by high-throughput sequencing in *Malus* X *domestica* Borkh. Mol. Biol. Rep. 41:5273. 10.1007/s11033-014-3399-8 24859975

[B72] Zamore P. D. Tuschl T. Sharp P. A. Bartel D. P. (2000). RNAi: double-stranded RNA directs the ATP-dependent cleavage of mRNA at 21 to 23 nucleotide intervals. Cell 101, 25–33. 10.1016/S0092-8674(00)80620-0 10778853

[B73] Zhang Y.-C. Yu Y. Wang C.-Y. Li Z.-Y. Liu Q. Xu J.. (2013). Overexpression of microRNA OsmiR397 improves rice yield by increasing grain size and promoting panicle branching. Nat. Biotechnol. 31, 848–852. 10.1038/nbt.2646 23873084

[B74] Zhou L. Chen J. Li Z. Li X. Hu X. Huang Y.. (2010). Integrated profiling of microRNAs and mRNAs: microRNAs located on Xq27.3 associate with clear cell renal cell carcinoma. PLoS ONE 5:e15224. 10.1371/journal.pone.0015224 21253009 PMC3013074

