# Supplementary figures and images for: microRNAs and Their Targets in Apple (Malus domestica cv. “Fuji”) Involved in Response to Infection of Pathogen Valsa mali
Source: Front Plant Sci. 2017 Dec 6;8:2081. doi: 10.3389/fpls.2017.02081 (PMC5723928; doi:10.3389/fpls.2017.02081)

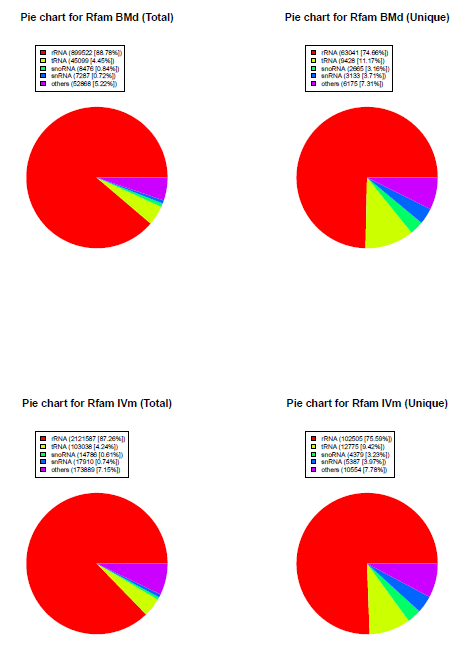

Supplement: Figure S1 — Rfam sequence category of BMd and IVm. [file Image1.TIF]

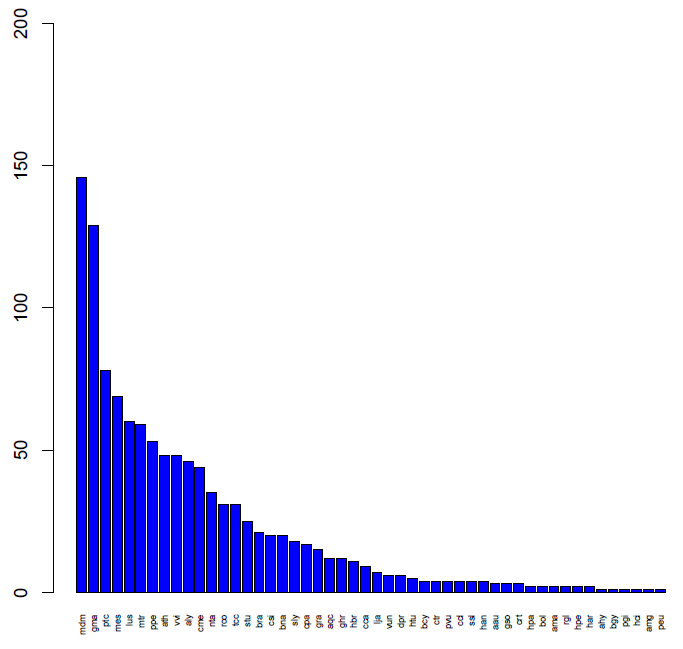

Supplement: Figure S2 — Conservation profile of the identified miRNAs. [file Image2.TIF]

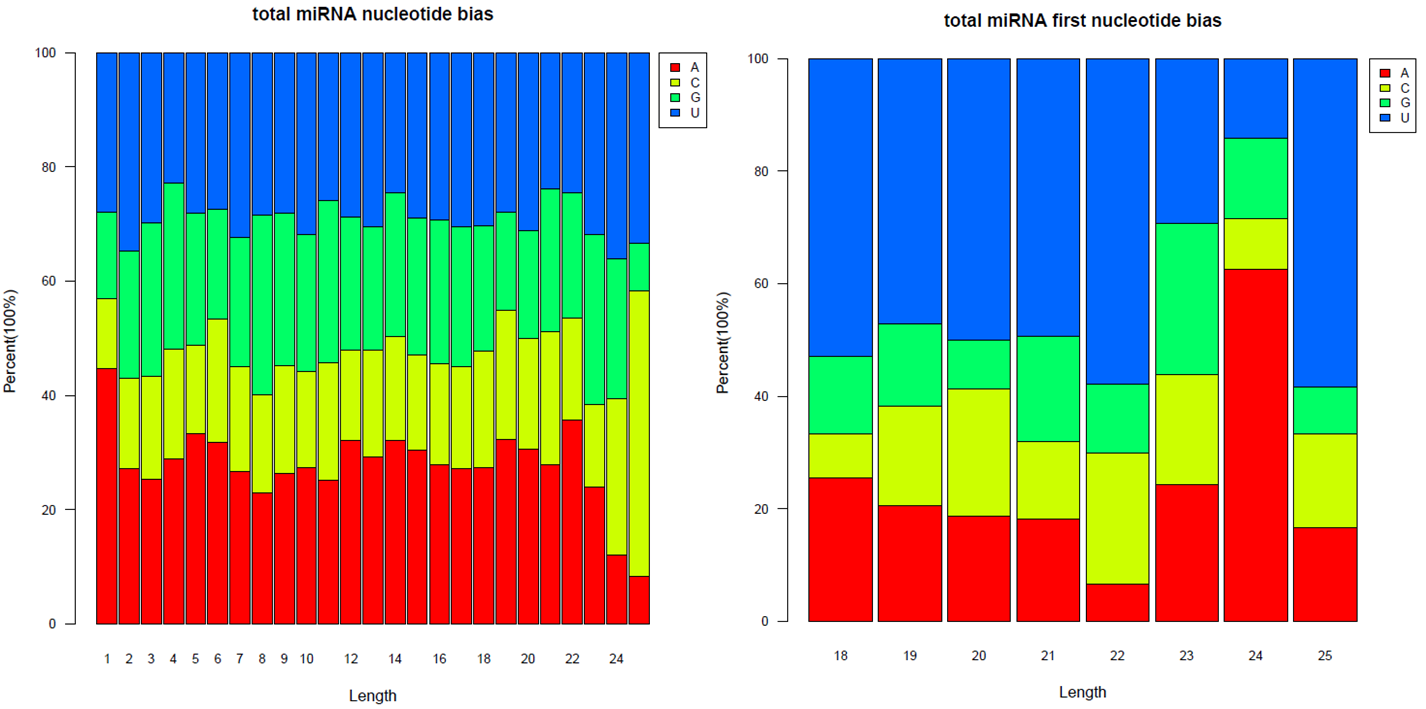

Supplement: Figure S3 — Nucleotide bias in each position of newly identified miRNAs. [file Image3.TIF]

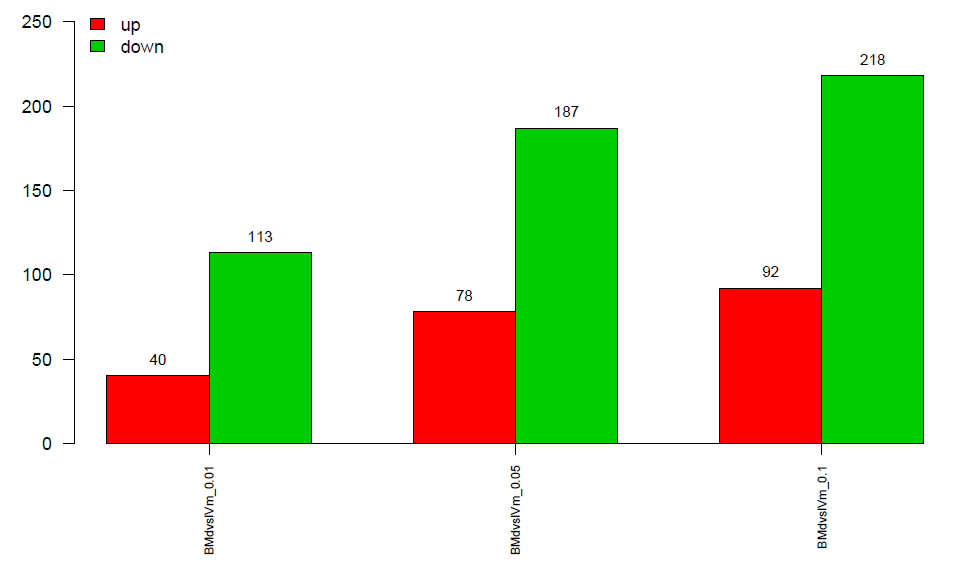

Supplement: Figure S4 — Barplot of differentially expressed miRNAs between IVm and BMd. [file Image4.TIF]

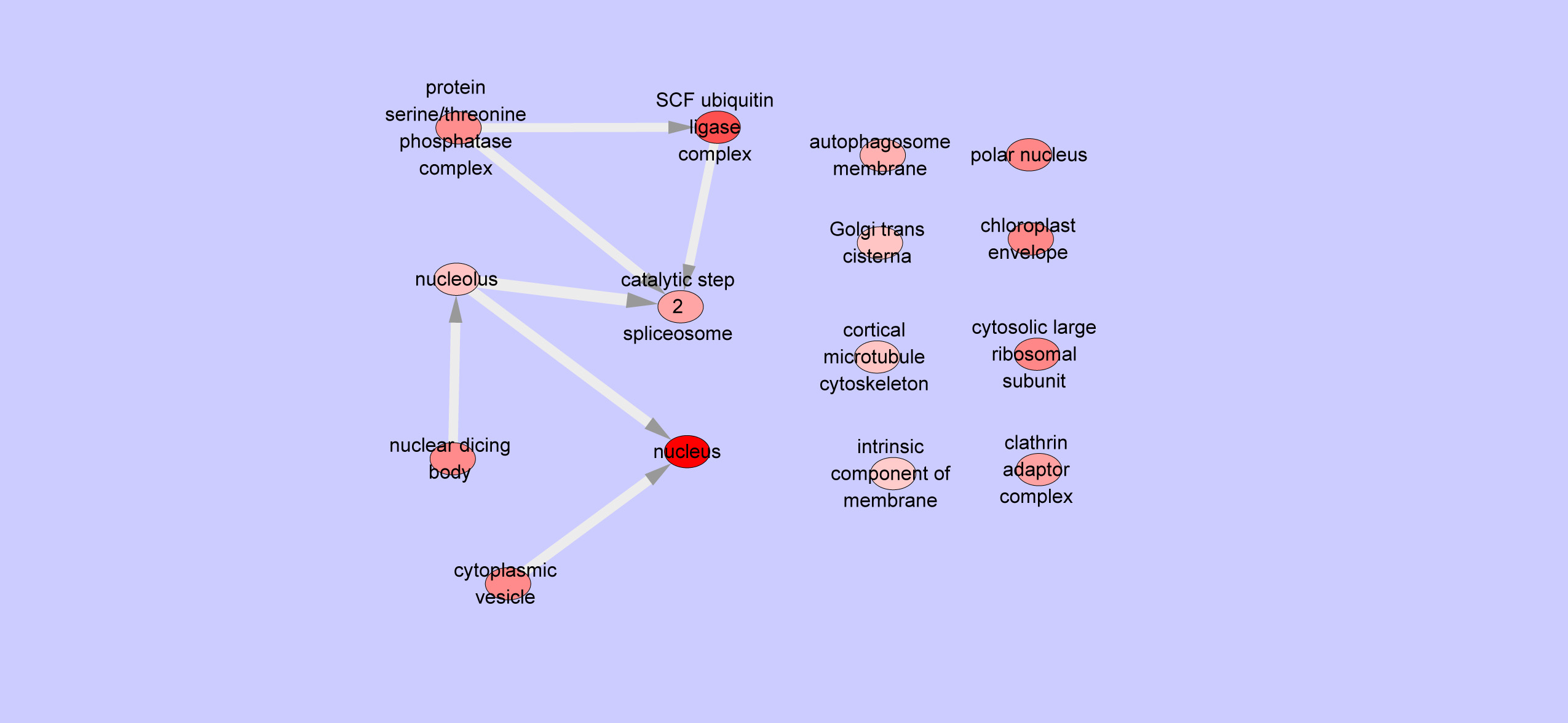

Supplement: Figure S5 — Enrichment analysis of target genes in “Cellular Component” category. [file Image5.JPEG]

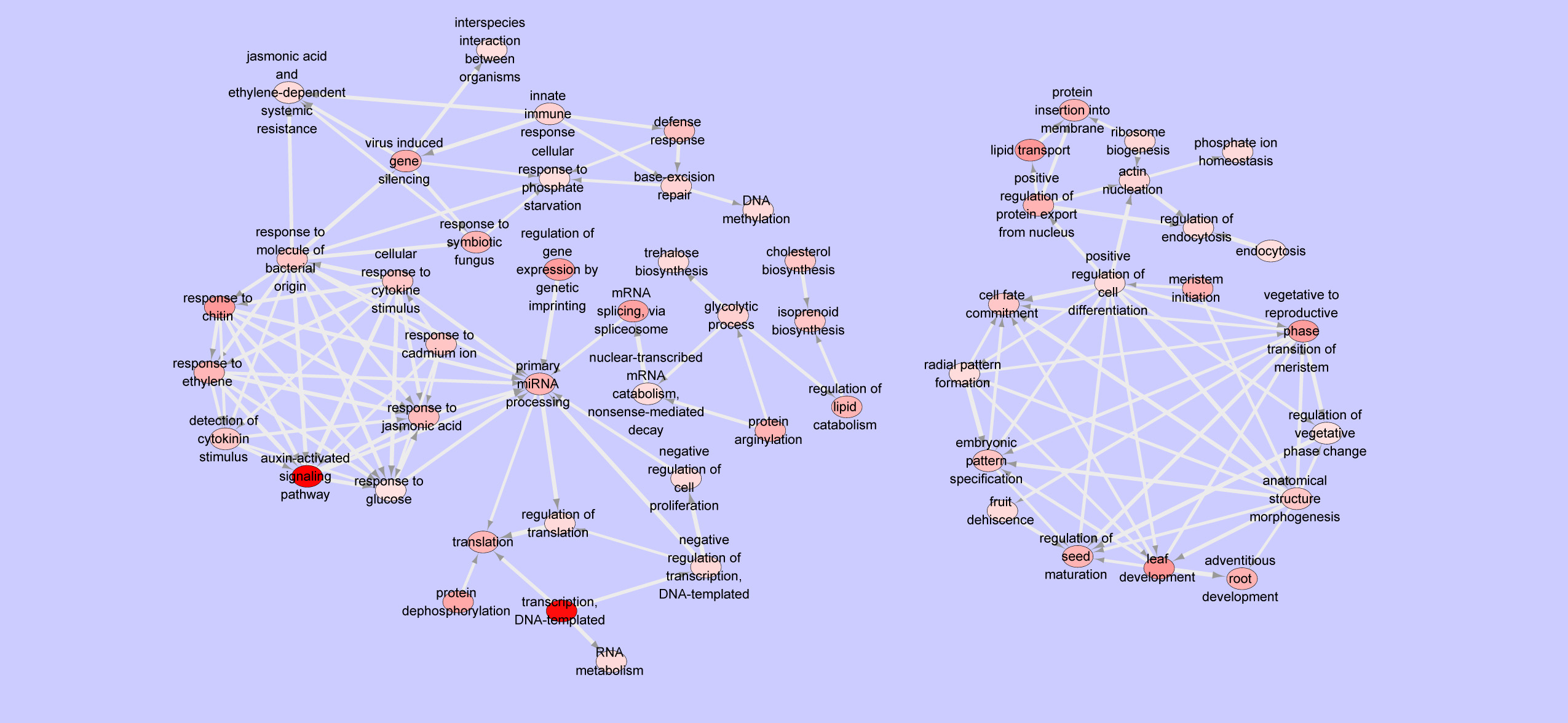

Supplement: Figure S6 — Enrichment analysis of target genes in “Biological Process” category. [file Image6.JPEG]

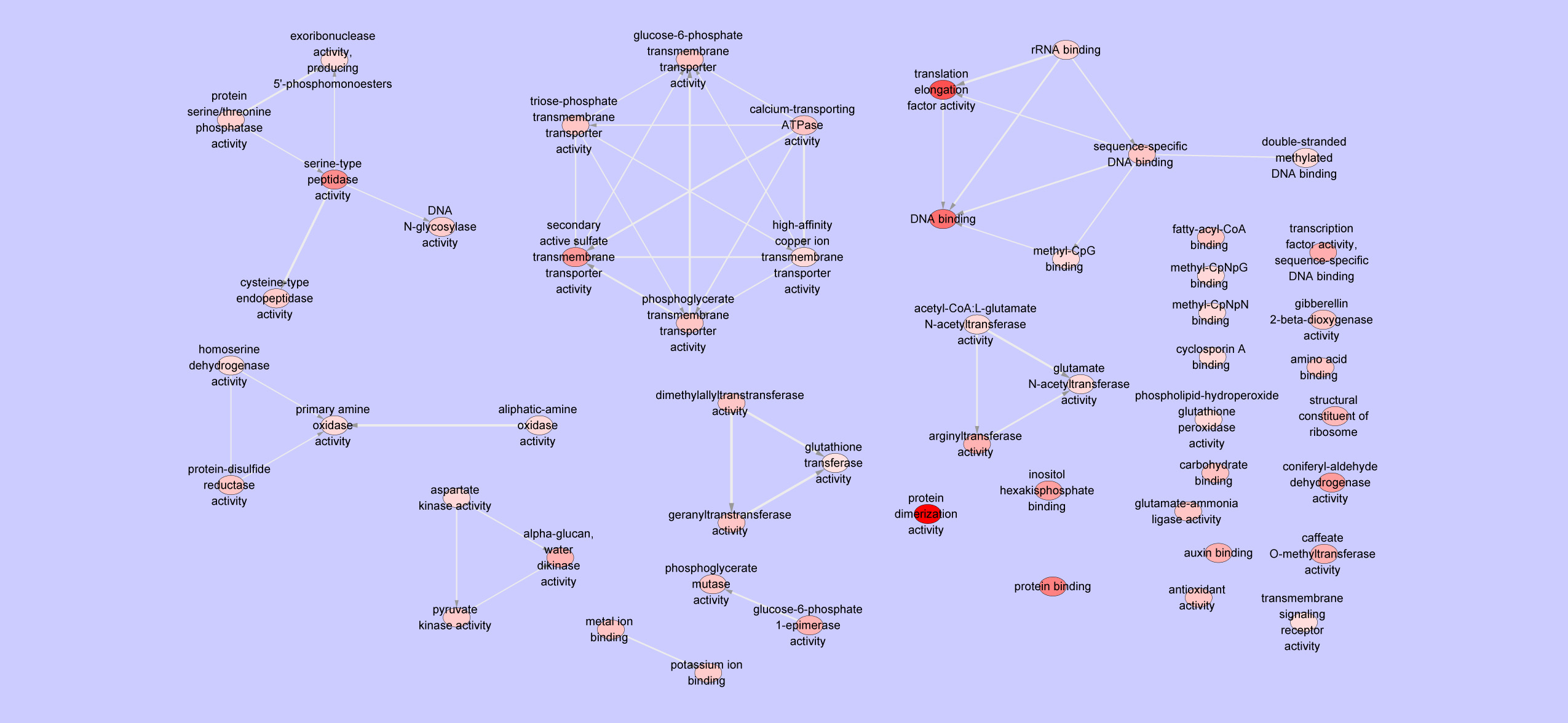

Supplement: Figure S7 — Enrichment analysis of target genes in “Molecular Function” category. [file Image7.JPEG]

# Statistics of Pathway Enrichment

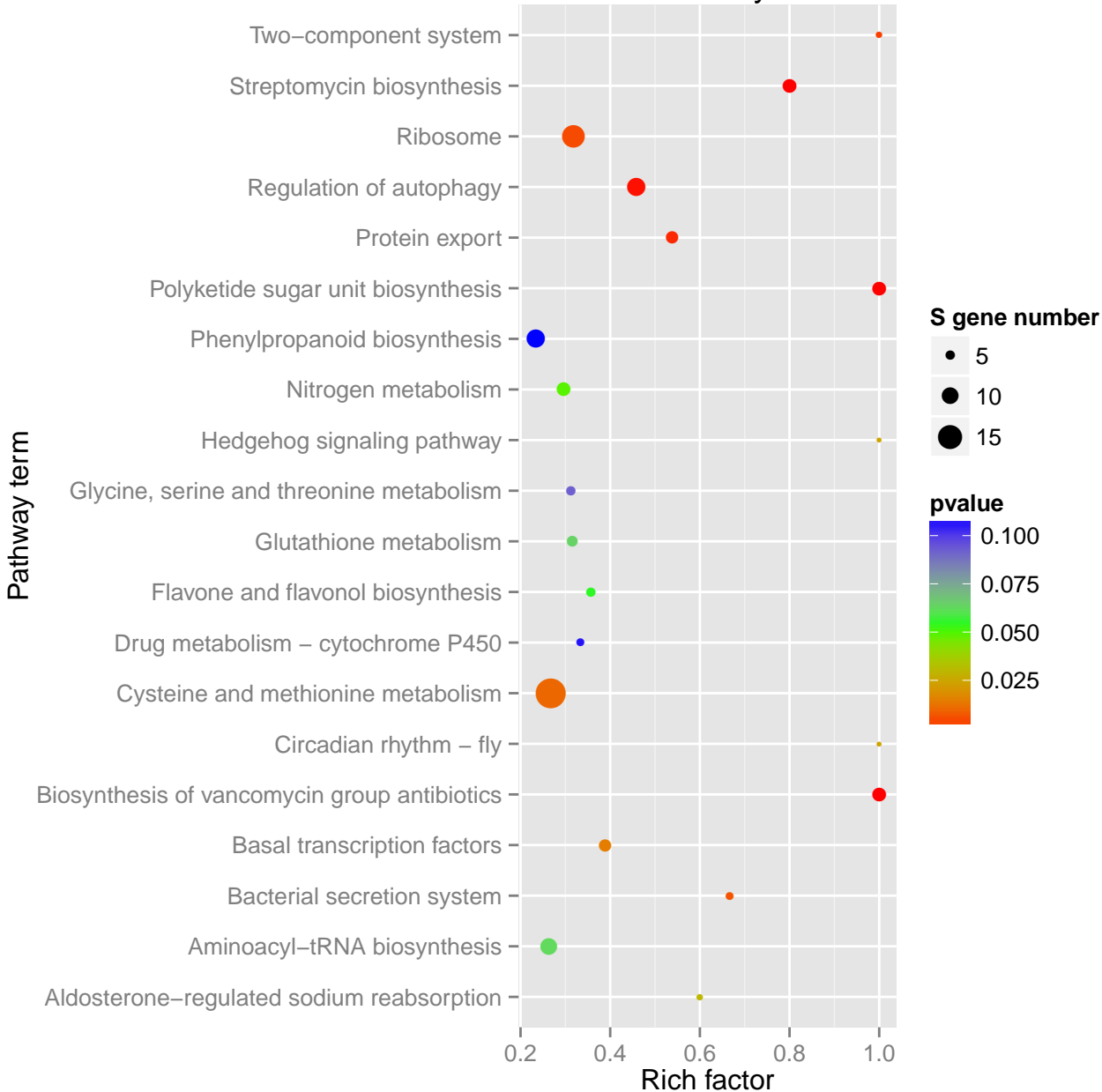

Supplement: Figure S8 — KEGG significant scatterplot between IVm and BMd. [file Image8.PDF]
