# Supplementary material for: microRNAs and Their Targets in Apple (Malus domestica cv. “Fuji”) Involved in Response to Infection of Pathogen Valsa mali
Source: Front Plant Sci. 2017 Dec 6;8:2081. doi: 10.3389/fpls.2017.02081 (PMC5723928; doi:10.3389/fpls.2017.02081)
Supplement: Table S8 — Relative expression of miRNAs in apple twig bark tissue - V. mali interaction. [file Table8.docx]

Table S8 Relative expression of miRNAs in apple twig bark tissue - *V. mali* interaction

| miRNA name | 12 hpi | 48 hpi |
| --- | --- | --- |
| mdm-miR482b | 1.30 | 0.25 |
| mdm-miR156a | 2.30 | 1.00 |
| mdm-miR319a | 0.36 | 0.51 |
| mdm-miR858b | 5.04 | 1.31 |
| mdm-miR391 | 30.67 | 0.99 |
| mdm-miR477a | 4.50 | 1.12 |
| mdm-miR535d | 0.98 | 0.97 |
| mdm-miR403a | 0.40 | 0.77 |
| mdm-miR164a | 1.12 | 4.26 |
| gma-miR6300 | 6.11 | 1.04 |
| bol-miR9410 | 0.99 | 1.31 |
| peu-miR2916-p3 | 0.74 | 1.45 |
| ppe-miR530 | 0.42 | 1.02 |
| gma-miR160-p3 | 0.49 | 0.63 |
| PC-5p-409096 | 0.98 | 0.89 |
| PC-3p-102462 | 0.67 | 0.72 |
| PC-3p-166024 | 0.90 | 1.25 |

According to the sequencing read number, 17 miRNAs were selected for transcript accumulation analysis in apple twig bark tissue after challenge with *V. mlali* (12 hpi and 48 hpi). The data were normalized to the expression level of apple translation elongation factor 1 alpha-subunit (EF). The relative expression level of the miRNAs in the *V. mali*-inoculated plants at each time point was calculated as the fold-change of the mock-inoculated plants at that time point using the comparative 2^-ΔΔ^CT method. The experiments were repeated with two independent biological replicates using newly extracted RNA and synthesized cDNA samples.
