# Supplementary material for: microRNAs and Their Targets in Apple (Malus domestica cv. “Fuji”) Involved in Response to Infection of Pathogen Valsa mali
Source: Front Plant Sci. 2017 Dec 6;8:2081. doi: 10.3389/fpls.2017.02081 (PMC5723928; doi:10.3389/fpls.2017.02081)
Supplement: Table S9 — Relative expression of target genes in apple twig bark tissue - V. mali interaction. [file Table9.docx]

Table S9 Relative expression of target genes in apple twig bark tissue - *V. mali* interaction

| Transcript number | Target anotation | 12 hpi | 48 hpi |
| --- | --- | --- | --- |
| XM_008392402.1 | ATL2 | 4.19 | 8.01 |
| XM_008386604.1 | RPPL1 | 2.90 | 2.18 |
| XM_008364202.1 | ATJ8 | 1.42 | 3.99 |
| XM_008375284.1 | RPM1 | 0.84 | 0.31 |
| XM_008351234.1 | SNC1 | 25.22 | 2.35 |
| XM_008344817.1 | At1g62630 | 0.27 | 2.02 |
| XM_008380985.1 | At3g14460 | 0.61 | 0.65 |
| XM_008379324.1 | RGA2 | 0.74 | 1.11 |
| XM_008354724.1 | RPP13L2 | 1.83 | 2.15 |
| XM_008379418.1 | At1g12290 | 0.47 | 0.49 |
| XM_008367951.1 | RGA3 | 2.54 | 1.58 |
| XM_008393337.1 | ACA10 | 0.82 | 0.73 |
| XM_008388371.1 | HRR25 | 1.41 | 3.03 |
| XM_008384095.1 | RD19A | 1.41 | 1.38 |
| XM_008363395.1 | BHLH80 | 0.82 | 0.59 |
| XM_008357865.1 | CIPK23 | 1.36 | 2.87 |
| XM_008386376.1 | FPS2 | 1.09 | 3.85 |
| XR_527149.1 | TAR1-A | 0.83 | 1.17 |
| XM_008384806.1 | NAC021 | 0.48 | 0.57 |
| XM_008372573.1 | UXS5 | 0.89 | 1.31 |
| XM_008391676.1 | WRKY75 | 7.05 | 16.26 |
| XM_008389442.1 | SF21 | 0.77 | 2.58 |

Twenty-two identified targets of miRNAs were selected for transcript accumulation analysis in apple twig bark tissue after challenge with *V. mali* (12 and 48 hpi). The data were normalized to the expression level of apple translation elongation factor 1 alpha-subunit (EF). The relative expression level of the miRNAs in the *V. mali*-inoculated plants at each time point was calculated as the fold-change of the mock-inoculated plants at that time point using the comparative 2^-ΔΔ^CT method. The experiments were repeated with two independent biological replicates using newly extracted RNA and synthesized cDNA samples.
